# Conservation of a microRNA cluster in parasitic nematodes and profiling of miRNAs in excretory-secretory products and microvesicles of *Haemonchus contortus*

**DOI:** 10.1371/journal.pntd.0006056

**Published:** 2017-11-16

**Authors:** Henry Y. Gu, Neil D. Marks, Alan D. Winter, William Weir, Thomas Tzelos, Tom N. McNeilly, Collette Britton, Eileen Devaney

**Affiliations:** 1 Institute of Biodiversity, Animal Health and Comparative Medicine, College of Medical, Veterinary and Life Sciences, University of Glasgow, Glasgow, United Kingdom; 2 Moredun Research Institute, Pentlands Science Park, Bush Loan, Penicuik, Edinburgh, United Kingdom; McGill University, CANADA

## Abstract

microRNAs are small non-coding RNAs that are important regulators of gene expression in a range of animals, including nematodes. We have analysed a cluster of four miRNAs from the pathogenic nematode species *Haemonchus contortus* that are closely linked in the genome. We find that the cluster is conserved only in clade V parasitic nematodes and in some ascarids, but not in other clade III species nor in clade V free-living nematodes. Members of the cluster are present in parasite excretory-secretory products and can be detected in the abomasum and draining lymph nodes of infected sheep, indicating their release *in vitro* and *in vivo*. As observed for other parasitic nematodes, *H*. *contortus* adult worms release extracellular vesicles (EV). Small RNA libraries were prepared from vesicle-enriched and vesicle-depleted supernatants from both adult worms and L4 stage larvae. Comparison of the miRNA species in the different fractions indicated that specific miRNAs are packaged within vesicles, while others are more abundant in vesicle-depleted supernatant. Hierarchical clustering analysis indicated that the gut is the likely source of vesicle-associated miRNAs in the L4 stage, but not in the adult worm. These findings add to the growing body of work demonstrating that miRNAs released from parasitic helminths may play an important role in host-parasite interactions.

## Author summary

Different species of parasitic worms release microRNAs, small non-coding RNA molecules, some of which are known to interact with host genes to alter the immune response. We characterized a cluster of four microRNAs from *Haemonchus contortus*, an important parasitic nematode of livestock. The miRNA cluster appeared to be present only in nematode worms that inhabit the intestinal tract and members of the cluster could be detected in the excretory-secretory products of adult *H*. *contortus*. Some of these miRNAs were also detected from the site of infection within the sheep host and from the draining lymph node suggesting that they may be released *in vivo* as well as *in vitro*. Analysis of excretory-secretory products released from adult worms and L4 larval stages demonstrated that specific miRNAs were contained within EV. These findings support a role for secreted miRNAs in the host-parasite relationship.

## Introduction

microRNAs (miRNAs) were first discovered in the free-living nematode *Caenorhabditis elegans* and have now been found in the cells of plants, animals and humans. These small RNAs are post-transcriptional regulators of gene expression and their dysregulation has been linked to a range of different pathologies including immune-related diseases [1] and cancer [2]. miRNAs act by binding to complementary sequences often located in the 3’UTR of target genes in the context of the RNA Induced Silencing Complex (RISC). This results in the degradation of the mRNA and/or repression of translation [3].

Parasitic nematodes, such as *Haemonchus contortus*, are closely related to *C*. *elegans* both belonging to nematode clade V [4]. By small RNA library sequencing and bioinformatics using available genome sequence data, we identified a total of 192 miRNAs from *H*. *contortus* L3 and adult worms in a previous study [5]. Of these, 44 were conserved between *H*. *contortus* and *C*. *elegans*, suggesting that the majority of *H*. *contortus* miRNAs were species-specific. *H*. *contortus* is an important parasite of small ruminants; it has a typical trichostrongyle life cycle in which sheep become infected by ingestion of infective third stage larvae (L3). These develop into L4 stages in the abomasum and then into adult worms, which have a blood-sucking habit resulting in anaemia and, in acute cases, death.

An interesting aspect of miRNA research stems from the finding that small RNA species can be secreted from cells and are detectable in the circulation [6–8]. A similar phenomenon has been observed for a number of parasitic helminths such as *Heligosomoides polygyrus* [9], *Dirofilaria immitis* [10], *Brugia malayi* [11] and *Schistosoma mansoni* [12]. In all these worms, miRNAs were shown to be released into the excretory-secretory (ES) products collected *in vitro*. In some cases, specific miRNAs were also detected in the serum of infected patients or animals suggesting that some helminth parasites release miRNAs *in vivo* as well as *in vitro* [13]. In an elegant study, Buck *et al*. [9] demonstrated that not only were miRNAs secreted by adult *H*. *polygyrus*, but that many were contained within exosomes, small cell-derived vesicles. Moreover, exposure of mouse cells to exosomes resulted in downregulation of host genes important for immune responses, such as *Dusp1* and *il-33r*. Those data provided the first example that secreted parasitic nematode miRNAs have the potential to regulate host immunity [9].

miRNAs are often found clustered in genomes and, in the original study of Winter *et al*., [5], eight *H*. *contortus* miRNA clusters containing 23 separate miRNAs were identified. *hco-miR-5352* was identified in that study and was shown to belong to a cluster of four miRNAs that appeared to be closely linked in the *H*. *contortus* genome. *hco-miR-5352* was of particular interest as comparative analysis showed that it was conserved in the gastro-intestinal nematode *Ascaris suum* but not in other clade III nematodes [5]. Here we provide a detailed characterisation of the *hco-miR-5352* cluster: we investigate its expression in different life cycle stages of *H*. *contortus*, its conservation in other parasitic nematodes and its presence in *H*. *contortus* excretory-secretory products (ES). Furthermore, we identify additional small RNA sequences in the ES products and EV of L4 and adult *H*. *contortus* and carry out comparative analysis with miRNAs within EV of the related parasitic nematode *Teladorsagia circumcincta*. The potential relevance of these miRNAs in the parasite life cycle is discussed.

## Methods

### Ethics statement

Experimental infections were carried out at the Moredun Research Institute, UK, as described previously [14]. All experimental procedures were examined and approved by the Moredun Research Institute Experiments and Ethics Committee (MRI E46 11) and were conducted under approved UK Home Office licence (PPL 60/03899) in accordance with the 1986 Animal (Scientific Procedures) Act, UK.

### Parasite material

Adult *H*. *contortus* of the strain MHco3(ISE) were harvested from sheep 28 days post-infection (p.i.) with 5000 infective L3 at the Moredun Research Institute. L4 were recovered at day 7 p.i. At post-mortem the abomasum was opened and washed with 0.9% saline solution to remove the worms, which were then extensively washed in saline solution.

The microarray was prepared by LC Sciences and contained 609 potential *H*. *contortus* miRNAs (not all of which were accepted by miRBase) and 238 *C*. *elegans* miRNAs present in miRBase release 15. The microarray was probed with RNA isolated from the following stages of *H*. *contortus*: sheathed L3, exsheathed L3 cultured for 24h at 37°C (activated L3), day 7 p.i. L4, adult males and females recovered 28 days p.i. and adult female gut tissue.

### RNA extraction and cDNA synthesis

RNA was extracted from whole worms, by grinding to a fine powder in a liquid nitrogen-cooled pestle and mortar before adding 1 ml of Trizol (Life Technologies) and proceeding exactly according to the manufacturer’s instructions. For host tissue, small pieces of abomasum or abomasal lymph node were collected at post-mortem from two sheep at 28 days p.i. with 5,000 L3, at which time male and female adult worms were present and the females were producing eggs. Equivalent tissue samples were harvested from two uninfected, pathogen-free sheep. Tissues were immediately added to tubes containing RNAlater. Care was taken to remove abomasal tissue from areas without any adult *H*. *contortus* present. Tubes were stored at -20°C, as recommended by the manufacturer, and RNA was isolated as required (see below).

30 mg of abomasal tissue or 10 mg of lymph node tissue were added to a hard tissue homogenizing CK28 tube (Bertin Instruments) and 1 ml of Trizol reagent added to each tube. Lymph node samples were homogenised using a Precellys 24 homogenizer (Bertin Instruments) for 2 x 50 second cycles at 6000 rpm, while abomasal tissues were homogenised for 5 x 23 second cycles at 6200 rpm, with 2 minutes on ice between each cycle. Following centrifugation at 14,000 rpm for 10 minutes at 4°C, 0.2 ml of chloroform was added per 1 mL of Trizol reagent and processing continued according to the manufacturer’s instructions. The final RNA pellet was resuspended in RNase free water and yield and purity assessed using a Nanodrop spectrophotometer.

PolyA tailing of RNA samples and cDNA synthesis was carried out using the miRNA 1st-Strand cDNA Synthesis Kit (Agilent Technologies) according to the manufacturer’s instructions. Samples were stored at -20°C.

### Semi-quantitative RT-PCR

RT-PCR was performed using the miRNA qRT-PCR Master Mix protocol (Agilent Technologies) on RNA extracted from whole adult worms, adult worm ES or abomasal and lymph node tissue from infected or uninfected sheep. Samples were analysed in the Agilent Mx3005P qRT-PCR System using MxPro QRT-PCR software (Agilent Technologies). All RT-PCR reactions were carried out in triplicate and mean values plotted using Microsoft Excel. Primer sequences are shown in S1 Table. For a normaliser miRNA for use with sheep tissues, the expression of three ovine miRNAs (*oar-miR-26a*, *oar-miR-103* and *oar-miR-122a*) was assessed. These were selected based on the expression of the bovine orthologues in a range of tissues [15]. *oaR-miR-26a* was selected as it could be amplified consistently from all ovine abomasal and lymph node samples tested.

### Parasite culture for excretory-secretory (ES) products

Approximately 100 adult worms (mixed males and females) or 250 L4 were cultured in 25 ml of RPMI supplemented with 1% glucose and 100 μg per ml penicillin/ streptomycin. Spent medium containing ES products was replaced with fresh medium every 24 hours for three days and was pooled as described below. ES was centrifuged at 1500 g for 5 minutes to remove released eggs and hatched L1 stages from adult ES or any debris from L4 ES, then filtered through a 0.22 μm filter and stored at -80°C. ES was concentrated from approximately 50 ml to 1 ml using Vivaspin 20 ml 10,000 MWCO sample concentrators and then stored at -80°C.

### Collection of extracellular vesicles and electron microscopy

Extracellular vesicles (EV) were collected by ultracentrifugation of 12.5 ml ES from adult worm or L4 cultures at 100,000 g for 2 h. The supernatant was aspirated carefully and retained (EV-depleted fraction). The final pellets were washed twice with PBS, re-suspended in 100–200 μl of PBS and stored at -80°C. In some experiments EV were processed for transmission electron microscopy exactly following the method of Tzelos *et al*., [16].

### RNA extraction and library preparation from ES and EV

RNA was extracted from total adult worm ES pooled from 24 and 48 h cultures for construction of a small RNA library at the University of Glasgow Polyomics Facility. In subsequent experiments, four additional libraries were constructed by LC Sciences using RNA isolated from EV or ES that had been depleted of EV, from cultures of adult worms or L4 stages. In the latter cases, ES was pooled from 24, 48 and 72 h cultures to optimize the amount of material available. In all cases, RNA was extracted using the Qiagen QIAzol system; 200 μl of concentrated ES or EV was added to 1 ml of QIAzol reagent and RNA extraction carried out according to the manufacturer’s instructions. RNA integrity was determined using an Agilent 2100 Bioanalyzer. All small RNA libraries were generated from RNA samples using the Illumina Trueseq™ Small RNA Preparation kit according to the manufacturer’s instructions and RNA sequenced using an Illumina GAIIx. For comparative purposes, concentrated EV was also obtained from the ES products of L4 stage of *T*. *circumcincta* and total RNA extracted as detailed above for *H*. *contortus*.

### Bioinformatic analysis

Homologues of the *hco-miR-5352* cluster were identified using BLASTN searches of WormBase ParaSite (http://parasite.wormbase.org/) with default search parameters. Sequences obtained from BLAST analysis were then aligned against the *H*. *contortus* sequence to determine pairwise similarity. Secondary structure prediction of the potential miRNA precursor sequences was carried out using Mfold 3.4 (http://mfold.rna.albany.edu). Sequence alignments were then carried out using Geneious version 6 (http://www.geneious.com [17]) using the following settings: Cost Matrix: 65% Similarity; Gap open penalty: 12; Gap extension penalty: 3; Alignment type: Local alignment (Smith Waterman)

Phylogenetic trees were created using Geneious with the settings as follows: Cost Matrix: 65% Similarity; Gap open penalty: 12; Gap extension penalty: 3; Alignment type: Local alignment (Smith Waterman); Genetic Distance Model: Tamura-Nei; Tree build Method: Neighbour-joining; Outgroup: no outgroup.

Illumina sequence reads from the total ES library were processed to clip adapter sequences and identify duplicate reads. miRDeep2 was used to map the reads to a list of miRNA precursor, mRNA, tRNA and rRNA sequences as described in Winter *et al*., [5].

Sequence reads from the EV and EV-depleted RNA libraries were mapped to *H*. *contortus* miRNAs from miRBase (version 21, 2014) and the *H*. *contortus* genome (h contortus.PRJEB506.WS248. genomic.fa.gz released on 04/09/2015) by LC Sciences, using a proprietary script. Reads that did not map to *H*. *contortus* were mapped to other nematode miRNAs in miRBase (namely *Caenorhabditis elegans*, *Caenorhabditis brenneri*, *Caenorhabditis briggsae*, *Ascaris suum*, *Pristionchus pacificus*, *Brugia malayi*, *Strongyloides ratti* and *Panagrellus redivivus*). The script aligned reads to the mature miRNA sequences and allowed for up to three mismatches in the 3’ end of the alignment. Mapped reads were normalised across the EV-enriched and EV-depleted libraries by calculating the geometric mean for each miRNA across the four samples. The raw reads were then adjusted based on the geometric means, allowing normalisation to miRNA read count. The median value of the adjusted read counts for each library were calculated (library size parameter) and the raw reads were then normalised based on the library size parameter. To identify reads mapping to the same list of precursor miRNA, mRNA, tRNA and rRNA sequences as described above and used by Winter et al., [5], Mapper in miRDEEP2 was used (https://www.mdc-berlin.de/36105849/en/research/research_teams/systems_biology_of_gene_regulatory_elements/projects/miRDeep [18]). This preformed a more stringent identification of *H*. *contortus* small RNAs than the initial LC analysis (no mismatches in the first 18 nucleotides).

Hierachical clustering was carried out using the matrix visualisation and analysis platform GENE-E version 3.0.240 (http://www.broadinstitute.org/cancer/software/GENE-E/) to identify groups of miRNAs with similar expression patterns from the various *H*. *contortus* ES libraries, both EV-enriched and EV-depleted. Data was normalised by scaling all numeric variables in the range [0,1] and normalised data imported into Gene-E. Row distance was calculated using the one-minus Pearson correlator metric, complete linkage and clustered by row. All other settings were default.

BLAST+ 2.4.0 was downloaded from NCBI and set up locally. RNA reads from the *T*. *circumcincta* library were set as queries for BLAST searching against the miRBase hairpin miRNA database (Release 21), with the BLAST parameters E-value being 0.01 and word-match size between the query and database sequences of 7.

Statistical analysis: Statistical significance was calculated using an analysis of variance test performed on MiniTAB Statistical Software version 16 (Minitab Inc, State College, PA, www.minitab.com).

## Results

### The *hco-miR-5352* cluster is most abundant in female worms

**Fig 1:**
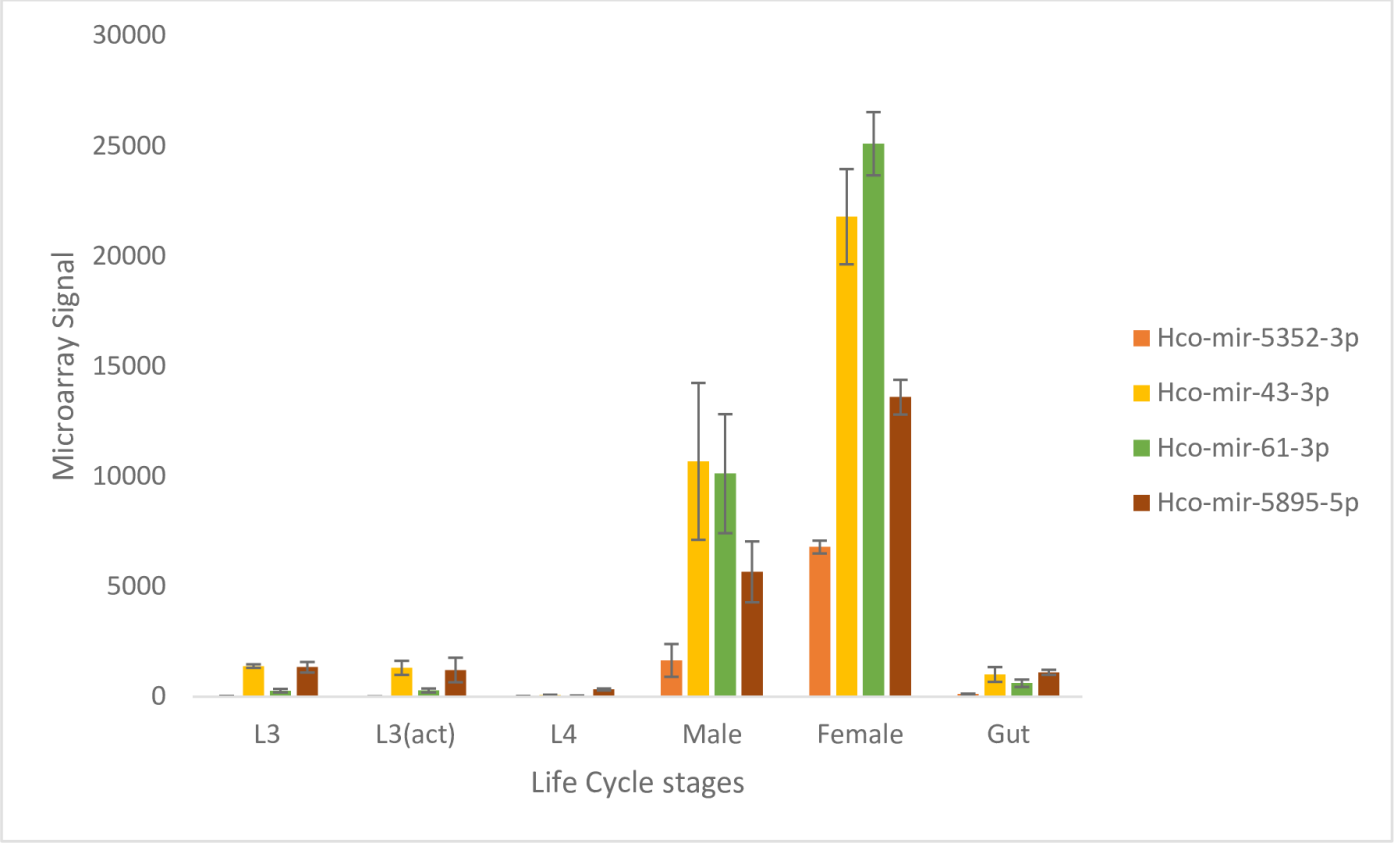
Developmental expression of the *Hco-miR-5352* cluster across the *H*. *contortus* life cycle as assessed by microarray. Data for sheathed L3, activated L3 (act) (exsheathed and cultured at 37°C for 24 hours), L4 stage collected 7 days p.i., adult males and females, collected at 28 days p.i. and gut tissue extracted from adult female worms. Microarray signals shown as the mean +/- SD of three biological replicates, except for L4 samples, where n = 2. Expression of the dominant arm is shown.

The *hco-miR-5352* cluster consists of four miRNA loci (*hco-miR-61*, *hco-miR-5352*, *hco-miR-43* and *hco-miR-5895*) all oriented in the same direction and closely linked in the genome, spanning a region of only 422 bp. The developmental expression of the miRNAs in the *hco-miR-5352* cluster was first investigated from a microarray containing all known *H*. *contortus* miRNAs. The array was probed with RNA from different life cycle stages of *H*. *contortus* and from gut tissue dissected from adult female worms. Minimal expression of the *hco-miR-5352* cluster sequences was observed in L3 stages (both sheathed L3 and activated L3, which had been exsheathed and cultured *in vitro*), the L4 stage and in adult female gut tissue. In contrast, all four miRNAs in the cluster were abundant in adult worms, with females showing higher levels of each miRNA compared to male worms (Fig 1), although this is only significant for *hco-miR-5352-3p* (*p*<0.05; log2>2.0).

Hierarchical clustering of the microarray expression data showed that the four miRNAs in the *hco-miR-5352* cluster group together based on their enrichment in adult female worms, suggesting that they may be co-expressed (see life cycle stage microarray). For all four miRNAs in the cluster, expression of both arms of the mature miRNA (3p and 5p arms) was identified in the original sequence data of Winter *et al*., [5]. The dominant miRNA from each hairpin was submitted to miRBase (3p for *hco-miR-61*, *hco-miR-5352* and *hco-miR-43* and the 5p for *hco-miR-5895*). Microarray data demonstrated that, while the expression of *hco-miR-43-5p* is much lower than the 3p arm, it is still expressed at a reasonable level in female worms (see microarray data).

### The *hco-miR-5352* cluster is conserved in clade V nematodes

**Fig 2:**
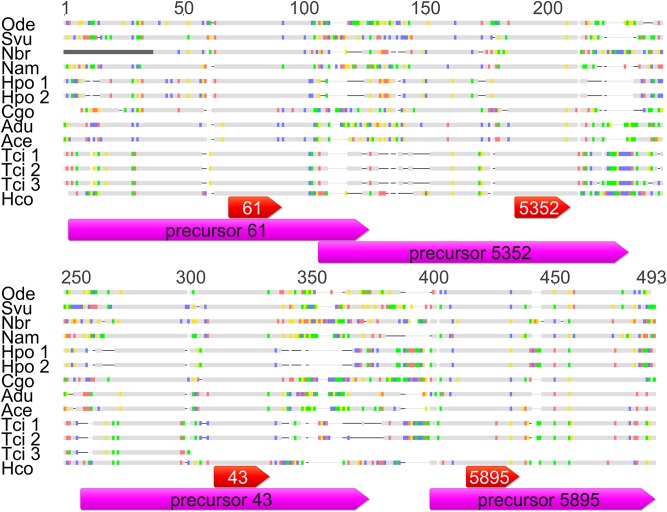
Alignment of the miRNA cluster sequences in clade V nematodes. BLAST searches were used to identify the scaffolds in WormBase ParaSite that were most similar to the *H*. *contortus miR-5352* cluster. Multiple sequence alignment was carried out with all sequences. Bases that are highlighted in colour show mismatches between the *H*. *contortus* miRBase sequence and the genome assembly; A-red, C-purple, T-green and G-yellow. Grey indicates conserved regions, black lines indicate gaps in the alignment. The magenta bars indicate the miRBase precursor sequence and the red bars indicate the miRBase mature sequence. Species names as follows: Ode, *Oesophagostomum dentatum*; Svu, *Strongylus vulgaris*; Nbr, *Nippostrongylus brasiliensis*; Nam, *Necator americanus*; Hpo, *Heligmosomoides polygyrus*; Cgo, *Cylicostephanus goldi*; Adu, *Ancylostoma duodenale*; Ace, *Ancylostoma ceylonensis*; Tci, *Teladorsagia circumcincta*; Hco, *Haemonchus contortus*. Multiple sequences were obtained for *T*. *circumcincta* and *H*. *polygyrus* as indicated by the number after the name.

BLASTN searches of the WormBase ParaSite database (parasite.wormbase.org) identified homologues of the *miR-5352* cluster in an additional ten clade V nematode species: *Teladorsagia circumcincta*, *Oesophagostomum dentatum*, *Nippostrongylus brasiliensis*, *Necator americanus*, *Strongylus vulgaris*, *Heligmosomoides polygyrus*, *Cylicostephanus goldi*, *Ancylostoma duodenale*, *Ancylostoma ceylanicum and Dictyocaulus viviparus*. The specific databases from which members of the *miR-5352* cluster were identified, the scaffold number and position and the location of these parasites within the host is shown in S2 Table. A multiple alignment of the cluster sequences from these clade V organisms along with the sequence of the *hco-miR-5352* cluster is presented in Fig 2. The *D*. *viviparus* cluster sequence is detailed below.

For all four miRNAs, there was significant identity between the precursor sequences across all nine species, ranging from 61.9 to 78.8% pairwise identity. The identity increased to over 95% when only the mature sequences were considered. All of these sequences were annotated manually using Geneious and RNA-fold and all form a stereotypical miRNA precursor hairpin loop.

From the BLAST searches, multiple sequences containing the *hco-miR-5352* cluster were identified from *H*. *polygyrus* and *T*. *circumcincta* (parasite.wormbase.org). From the contig location coordinates, *T*. *circumcincta* sequences 1 and 2 were located at 144782–144965 and 144994–145200 of contig 190 while *H*. *polygyrus* sequences were also located close together at 48067–48260 and 48281–48489 on scaffold 0001328. Both sets of sequences showed near 100% identity to each other, suggesting that they may have arisen from recent duplication, although it is also possible their identification in regions of close proximity may result from poor assembly of these genomic regions. *T*. *circumcincta* sequence 3 was located on a different contig and was shorter (see S1 Fig).

*Asu-miR-5352* is the only other miRNA in miRBase designated as *miR-5352* and the precursor miRNA shows 70% identity to the *H*. *contortus* sequence (S2 Fig) [19]. Six versions of *Ascaris miR-43* were identified in miRBase, showing 69% identity *to hco-miR-43*, however, *Ascaris miR-61* and *miR-5895* were not identified in the current version of miRBase. *hco-miR-5352* cluster precursor and mature sequences, along with *A*. *suum miR-43* and *5352* sequences, were aligned to both *A*. *suum* and *Ascaris lumbricoides* scaffolds that contained *miR-5352*. The mature *Ascaris* miRNA sequences were very similar, differing by only one base (99.8% pairwise similarity) as shown in S2 Fig. The *Ascaris* miRNAs were organised in the same order as in *H*. *contortus*, albeit with a much larger gap between the two precursors. *Asu-miR-5352* was then used to screen for *miR-5352* sequences in other clade III species, revealing their presence only in other ascarid parasites. *miR-5352* and *miR-43* were identified from *Toxocara canis*, while *Anisakis simplex* contained only *miR-5352*.

In addition to the clade III species, the mature *miR-5352* sequence was also identified from the bovine lungworm *Dictyocaulus viviparus*. The region of alignment was 23 bp long and was 95.7% similar to the *H*. *contortus* mature sequence (S3 Fig). Further searches identified a 100% match to the mature *hco-miR-43* and a 90.9% match to the mature *hco-miR-61* sequence. From the location, the three matches described above were clustered together. In depth analysis of the surrounding regions identified a sequence aligning to *hco-miR-5895*, with 87.9% identity. Mfold analysis of the predicted precursor sequences indicated that the *D*. *viviparus* miRNAs were able to fold into hairpin loops (S4 Fig).

A phylogenetic tree using the sequences of the *miR-5352* cluster from WormBase ParaSite is shown in S5 Fig, demonstrating that the cluster in *A*. *suum* and *D*. *viviparus* is more distantly related to that in the other species. From all the parasitic nematodes for which genome data is available, only worms from the sub-order Strongylida contained the complete *hco-miR-5352* cluster, with the exception of the super-family Metastrongyloidea. Neither *Angiostrongylus costaricensis* nor *A*. *cantonensis* contained any member of the *hco-miR-5352* cluster

### *Hco-miR-5352* can be detected in excretory-secretory (ES) products and in infected sheep tissues

**Fig 3:**
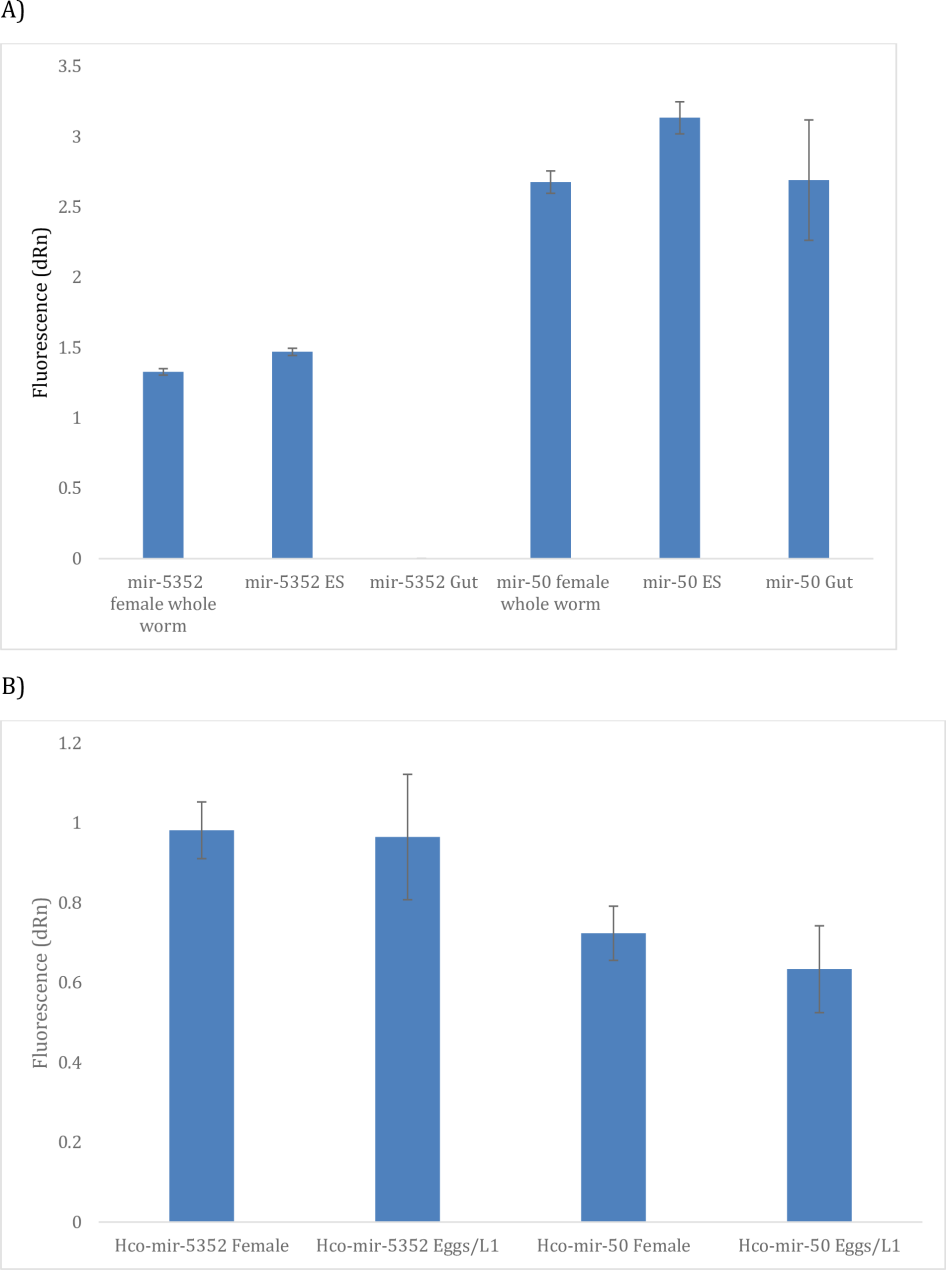
(A) qRT-PCR amplification of *Hco-miR-5352* in adult female whole worms, adult ES and adult gut tissue. Results shown represent the mean ± SD of three technical replicates. *Hco-miR-50* was used as a control gene for PCR amplification. Raw qRT-PCR readings are shown as no miRNA was known that could be used to normalise expression across all samples. (B) qRT-PCR amplification analysis of *Hco-miR-50* and *Hco-miR-5352* expression in adult females and egg/L1 sample. Results shown represent the mean ± SD of three technical replicates, with *Hco-mir-50* shown as a control for PCR amplification as used in Fig 3A.

As the *miR-5352* cluster appeared to be expressed only in worms that reside at mucosal surfaces, we next determined whether it may be secreted from the parasites and could be detected in parasite ES or in infected tissues. Mixed sex adult *H*. *contortus* were cultured *in vitro* and ES collected over a 48 h period. RNA was extracted from concentrated ES and qRT-PCR carried out to detect *miR-5352*, with whole adult female *H*. *contortus* cDNA being used as a positive control. In addition, several worms were removed prior to *in vitro* culture and were dissected to remove the worm intestine, from which RNA was also prepared. Fig 3A presents the analysis of *hco-miR-5352* expression in adult female whole worms, adult ES and adult gut samples. qRT-PCR showed that *hco-miR-5352* could be detected in the ES products and female worm extract but not in gut tissue, consistent with microarray data. While it was not possible to quantify the *hco-miR-5352* signal precisely, a constitutively expressed miRNA, *hco-miR-50*, could be amplified from all three samples.

As the miRNAs in the *hco-miR-5352* cluster were enriched in adult female worms, but not expressed in the adult female gut tissue, we assayed the presence of *hco-miR-5352* in eggs/L1 collected from *in vitro* cultured worms. *miR-5352* could be detected in eggs/L1 by qRT-PCR at a similar level to that of the adult female (Fig 3B).

**Fig 4:**
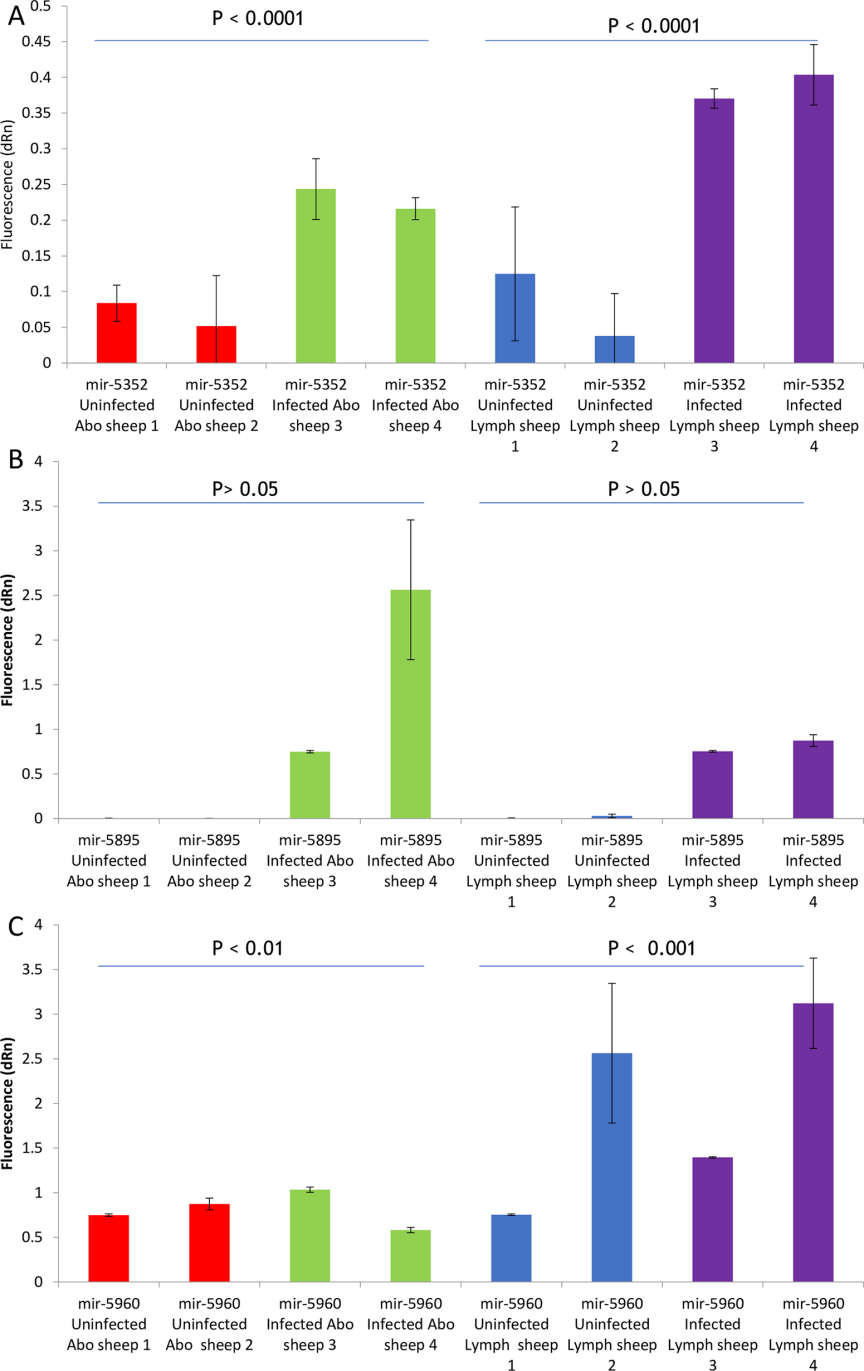
qRT-PCR to detect presence of A) *Hco-miR-5352*, B) *Hco-miR-5895* and C) *Hco-miR-5960* in two infected and two pathogen-free (uninfected) sheep from abomasal (abo) and draining lymph node (lymph) tissue. Samples were obtained from the abomasum and lymph nodes and qRT-PCR was carried out in triplicate. Mean values are shown with error bars representing the SD. Fluorescence values were normalised to *oar-miR-26a* qRT-PCR. P values shown were calculated using an analysis of variance test.

To determine whether *Haemonchus* miRNAs could be detected in tissue from infected sheep, indicative of the release of parasite miRNAs *in vivo*, RNA was extracted from small pieces of abomasal tissue or from the abomasal lymph nodes dissected from *H*. *contortus* infected and pathogen-free sheep at post-mortem. Two members of the cluster, *hco-miR-5352* and *hco-miR 5985*, were selected on the basis of their lack of similarity to sheep miRNAs, while *hco-miR-5960* was also assayed, as it is the most abundant *Haemonchus* miRNA in ES material (see below). The data in Fig 4 shows the signal obtained from infected and uninfected tissues for all three miRNAs normalised to *oar-miR-26a* (see Materials and Methods and S6 Fig). For abomasal tissue samples and particularly the lymph node, *hco-miR-5352* could be detected at a greater level in infected relative to uninfected animals (P<0.0001), while *hco-miR-5895* gave no signal from tissue from uninfected animals but was detected in infected tissue samples (P<0.001). In contrast, levels of *hco-miR-5960* showed no significant difference between infected and uninfected tissues. Interestingly for *hco-miR-5352*, the signal from the draining lymph node in infected sheep was significantly higher than that from the abomasal tissue (P = 1.59E-05).

### *H*. *contortus* ES contains multiple additional small RNA sequences

The data presented above demonstrated that *hco-miR-5352* could be detected in adult worm ES material using qRT-PCR. In order to characterise the complement of small RNA species present in *H*. *contortus* ES, a small RNA library was constructed from adult worm ES. Over 15 million reads were sequenced and mapped to previously identified *H*. *contortus* RNA sequences [5]. Alignments were accepted if the read was longer than 18 base pairs and had no more than one mismatch.

The initial study of Winter *et al*., [5] identified 462 potential miRNA sequences from adult whole worm and L3 stages of *H*. *contortus*, of which 211 were submitted to miRBase and 192 accepted [5]. This number has been revised in the current version of miRBase (release 21, http://www.miRBase.org/, accessed 10/01/2015) and now consists of 195 miRNAs. Of these 195 accepted *H*. *contortus* microRNAs, 85 were found in the adult ES small RNA library. In addition, a further 83 sequences were identified that mapped to miRNAs that had been rejected from the miRBase list for a variety of reasons including: not folding according to miRBase programs or the Computational Identification of microRNA program (CID-miRNA) [20], similarity to existing rRNA or tRNA sequences, or low counts (<10 reads) in the adult library.

**Fig 5:**
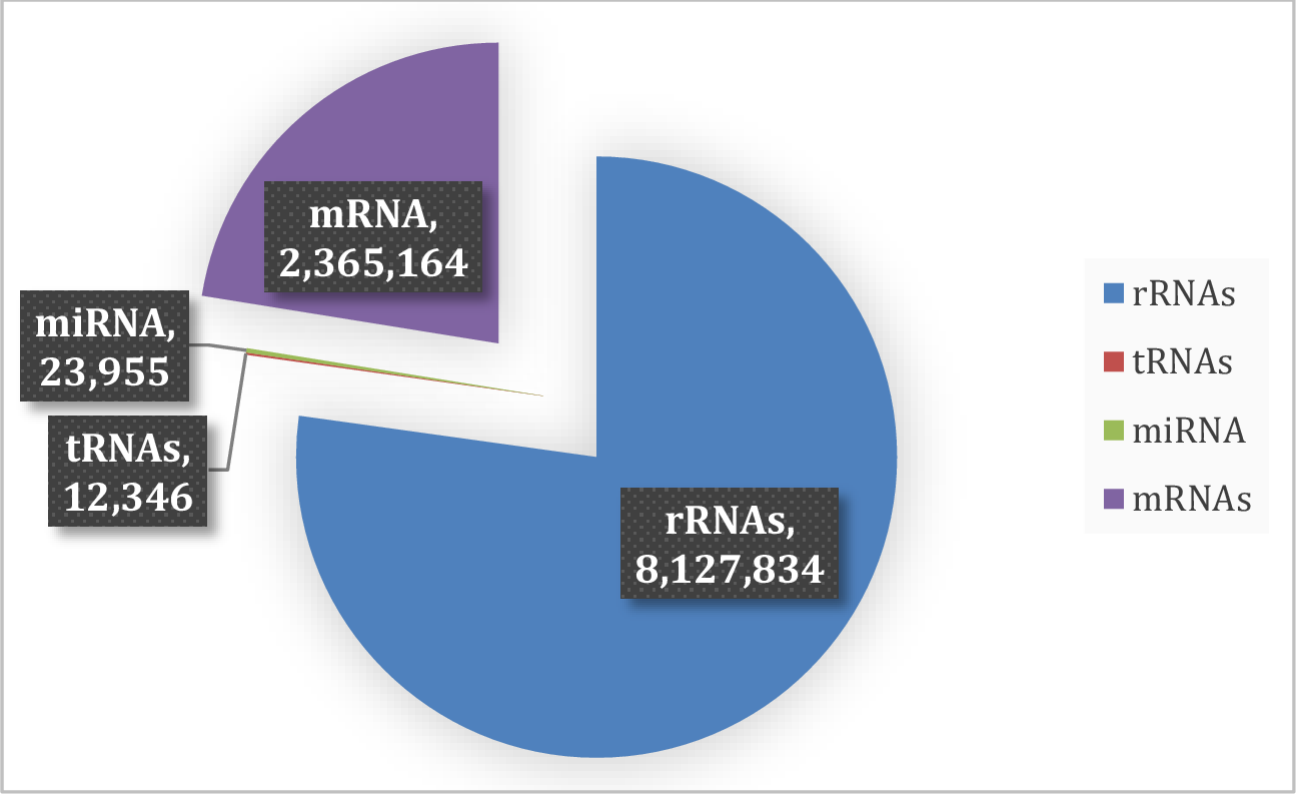
Composition of RNA reads from the *H*. *contortus* adult ES small RNA library mapped to *H*. *contortus* RNA sequences. Numbers indicate number of unique reads for each RNA category.

All reads from both the adult whole worm and adult ES small RNA libraries were mapped to all 462 potential miRNA sequences as well as to tRNA, rRNA and mRNA sequences obtained by Winter *et al*., [5]. In the ES small RNA library, 77% of the total reads mapped to ribosomal RNA sequences, with mRNAs being the second most abundant class with 22% of the reads (Fig 5). Transfer RNAs (tRNAs) comprised the smallest group (0.1%) with miRNAs representing 0.2% of the reads. In comparison, in the adult whole worm small RNA library, rRNAs made up 32% of the reads, followed by miRNAs (14%). Reads from tRNAs constituted the smallest group (4%) with mRNAs comprising 14% [5].

Due to the difference in total read numbers between the adult ES library and adult worm extract library, the miRNAs were ranked by the number of reads. Tables 1–3 show the ten most abundant miRNA sequences for the adult whole worm and adult ES libraries, including miRNA sequences that were not accepted by miRBase. Five of the ten most abundant sequences in the adult ES library (Table 1) are not accepted miRNAs; in the adult whole worm library, only *hco-0325* is not an accepted miRNA. *hco-0129*, *hco-0135* and *hco-0134* were rejected as miRNAs due to their low number (<10 reads) in the adult whole worm library. However, in the ES library, their abundance is fourth, fifth and sixth respectively and all show read numbers >500. These results demonstrate that there are differences between adult whole worm and adult ES libraries and that there are sequences in the ES that are likely to be novel miRNAs, although their expression is low in adult whole worm libraries.

**Table 1:** Top 10 miRNAs, ranked by total reads, found in the adult ES small RNA library.

Rank in adult ES library	miR name	Number of reads	Rank in adult whole worm library
**1**	*hco-miR-5960*	16743	55
**2**	hco0325*	2401	9
**3**	*hco-miR-5895*	1093	4
**4**	Hco0129*	738	347
**5**	Hco0135*	700	354
**6**	Hco0134*	503	328
**7**	Hco0324*	433	27
**8**	*hco-miR-61*	406	1
**9**	*hco-miR-45*	403	22
**10**	*hco-miR-228*	393	13

* indicates sequences that were discarded from the original paper (i.e. have no miRBase name)

**Table 2:** Top 10 miRNAs ranked by total reads in the adult whole worm library [5].

Rank in adult extract library	miR name	Number of reads	Rank in ES library
**1**	*hco-miR-61*	543826	8
**2**	*hco-miR-71*	54876	24
**3**	*hco-miR-5983*	47494	53
**4**	*hco-miR-5895*	25614	3
**5**	*hco-miR-84*	19745	102
**6**	*hco-miR-5884*	18952	17
**7**	*hco-miR-43*	18561	11
**8**	*hco-miR-5352*	15943	12
**9**	Hco0325*	12689	2
**10**	*hco-miR-87b*	12230	#N/A

* indicates sequences that were discarded from the original paper (i.e. have no miRBase name)

**Table 3:** Comparison of the top 10 miRBase miRNAs in the adult ES library and their corresponding ranking in the adult whole worm library.

microRNA	Rank in adult ES library	Rank in adult whole worm library
*hco-miR-5960*	1	48
*hco-miR-5895*	2	4
*hco-miR-61*	3	1
*hco-miR-45*	4	21
*hco-miR-228*	5	12
*hco-miR-43*	6	7
*hco-miR-5352*	7	8
*hco-miR-5938*	8	139
*hco-miR-40b*	9	31
*hco-miR-5885c*	10	15

The miRNAs are ranked by total normalised reads.

Three of the top ten most abundant miRNAs in the adult ES library, *hco-miR-61*, *hco-miR-5895* and *hco-miR-0325*, also appear in the top ten list for the adult whole worm library. *hco-miR-87b*, on the other hand, is the tenth most abundant miRNA in the adult whole worm library but does not appear at all in the ES library (Table 2). The large variation in the relative abundance of these miRNAs suggests that there is selective release of miRNAs in the ES. Table 3 shows a comparison of the rankings showing only the miRBase-accepted miRNAs ranked by total normalised reads.

The miRNAs of the *hco-miR-5352* cluster are highly abundant in both libraries and show similar rankings, with expression of *hco-miR-5352* being the lowest of the four, followed by *hco-miR-43*. In the whole worm adult library, *hco-miR-61* expression is higher than that of *hco-miR-5895*, but in the ES library, the reverse is true.

### *H*. *contortus* ES products contain extracellular vesicles

**Fig 6:**
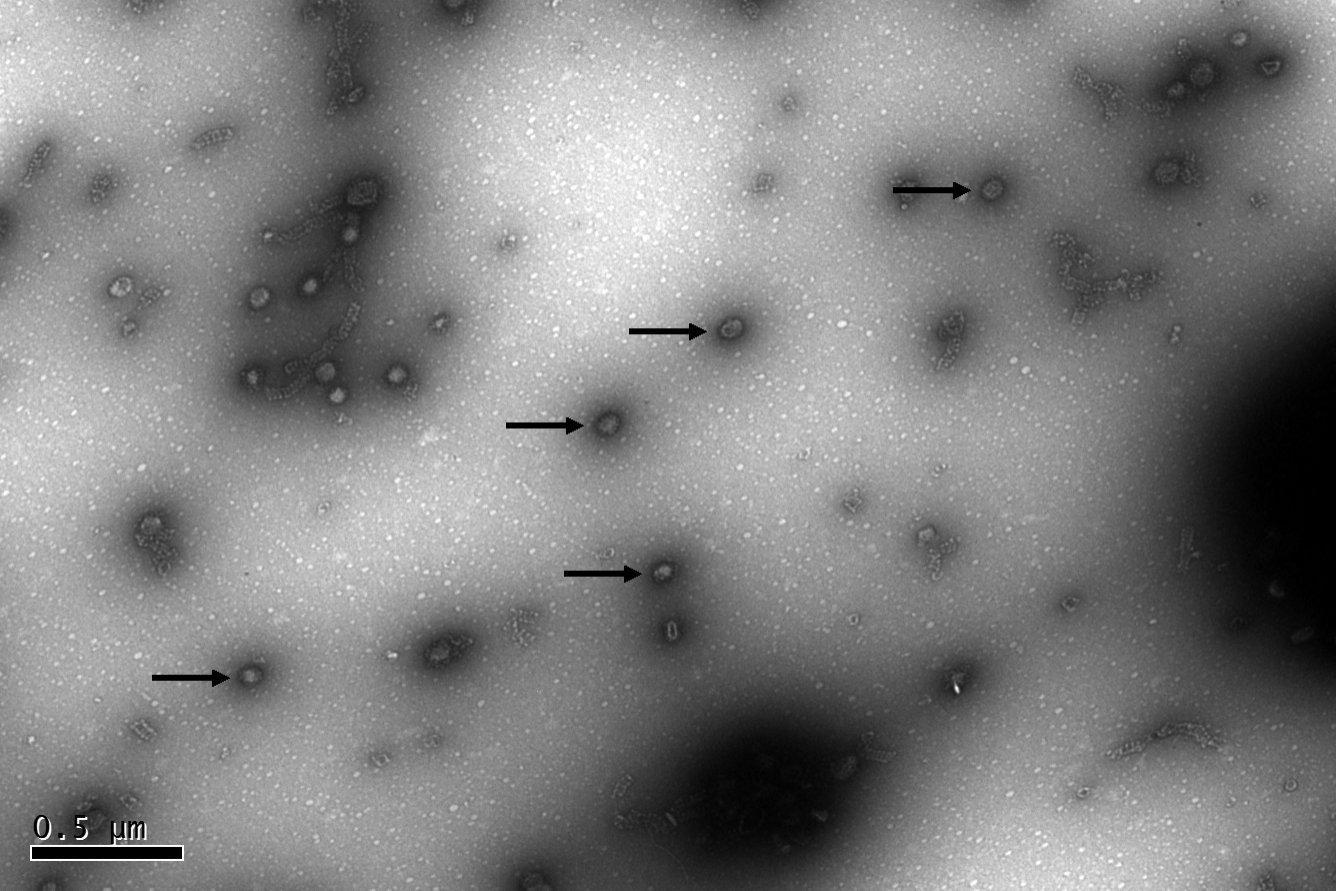
Transmission electron microscopy of ultracentrifugation pellet (0.7 μg/μl total protein) from adult *H*. *contortus* ES products. Scale indicates 500 nm. Black arrows indicate extracellular vesicle-like structures.

miRNAs have previously been shown to be present in both the ES supernatant and in EV purified from nematode ES products [9]. To determine whether *H*. *contortus* ES contains EV, adult worm ES was collected, ultracentrifuged and processed for transmission electron microscopy. A large number of small circular structures were identified of a size consistent with EV, as shown in Fig 6. These structures were similar in appearance to those identified in both *H*. *polygyrus and T*. *circumcincta* ES [9,16].

### Comparative analysis of small RNA libraries prepared from EV-enriched and EV-depleted supernatant from *H*. *contortus* ES

To further investigate the RNA content of *H*. *contortus* ES, four additional small RNA libraries were generated using ES from adult worms or L4 that had been separated into either EV-enriched or EV-depleted fractions. The number of total reads obtained from each of these samples were lower than that from the unfractionated adult ES RNA library described above. In total, the four libraries contained over 23 million reads that mapped to the *H*. *contortus* genome. Table 4 shows the proportion of different RNA species mapping to known *Haemonchus* RNAs, as defined in Winter et al., [5]. For the L4 stage, the EV-depleted library showed enrichment of miRNA reads, which was not observed with the L4 EV-enriched library, nor in the adult libraries.

**Table 4:** Composition of small RNA libraries prepared from EV-enriched or -depleted ES from L4 or adult stages, mapped to known *H*. *contortus* RNA species.

	Mappable reads	Number of reads mapping to Hco RNA lists	microRNA	rRNA	tRNA	mRNA
**L4 EV-enriched**	3,321,791	32,079	12,320 (38.4%)	16,046 (50%)	301 (1%)	3,412 (10.6%)
**L4 EV depleted**	5,822,406	183,955	123,726 (67.2%)	47,667 (25.9%)	3,041 (1.7%)	9,521 (5.2%)
**Adult EV-enriched**	9,616,116	408,290	66,091 (16.2%)	273,292 (66.9%)	1,740 (0.4%)	67,167 (16.5%)
**Adult EV depleted**	5,962,233	3,709,814	52,889 (1.4%)	2,991,849 (80.7%)	29,896 (0.8%)	635,180 (17.1%)

Number and percentage of normalised reads representing different classes of *H*. *contortus* RNAs only (as identified by Winter et al [5]) are shown.

**Fig 7:**
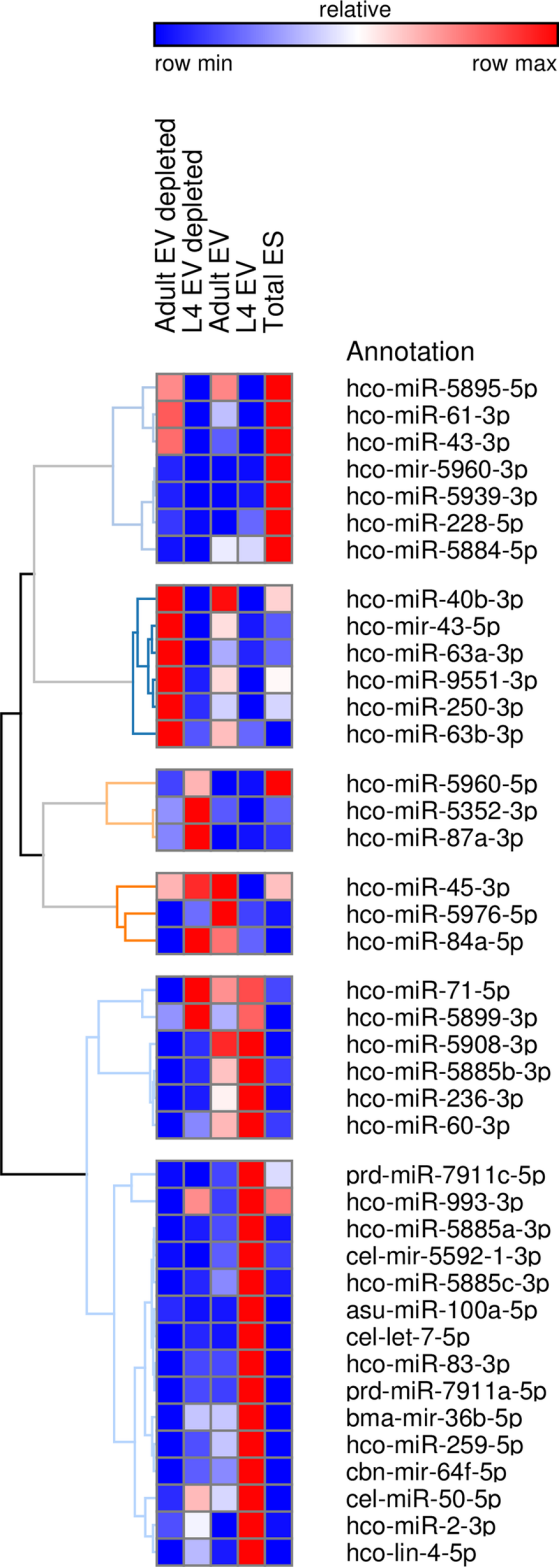
Hierarchical clustering of microRNA reads in the normalised small RNA libraries prepared from EV-enriched or EV-depleted ES from *H*. *contortus* adults and L4, or from total ES from adult worms. List of miRBase microRNAs identified in the small RNA libraries. microRNAs that had less than 10 reads in two or more of the libraries were discarded from the final list. Blue boxes indicate low number of reads, white indicates medium expression and red boxes indicate high number of reads.

Reads were also mapped to nematode sequences in miRBase version 21 and to the *H*. *contortus* genome by LC Sciences, as described in Methods. S3 Table shows the normalised reads for the miRNAs in the EV and EV-depleted supernatant libraries mapped to all nematode miRNAs in miRBase. This list also includes star strand miRNAs, such as *hco-miR-5960-3p*, which, while not specifically stated in the miRBase database, can be inferred from the precursor sequence. Using these data, an analysis of the abundance of specific miRNAs in EV-enriched versus EV-depleted ES material was carried out. Some miRNAs, such as the cluster member *hco-miR-5895-5p*, were present at similar levels in both adult EV and EV-depleted libraries, as were *hco-miR-5899-3p* and *hco-miR-40b-3p*. In contrast, other miRNAs were abundant in adult EV but had negligible read counts in the adult EV-depleted libraries (see S3 Table). These data are presented by hierarchical clustering in Fig 7 and demonstrate the enrichment of specific miRNAs in different ES libraries. In particular, this analysis highlights the relative abundance of certain miRNAs in the L4 EV-enriched library. In the L4 libraries, *hco-miR-5885a-3p*, *hco-miR-5885b-3p*, *hco-miR-5885c-3p*, *hco-miR-5908-3p*, *hco-lin-4-5p*, *hco-miR-83-3p* and sequences homologous to *cel-let-7-5p* and *asu-miR-100a-5p*, not previously identified from *H*. *contortus*, are all highly expressed in the L4 EV-enriched and have lower read counts in the L4 EV-depleted libraries. This is consistent with data on secreted miRNAs of *H*. *polygyrus* that showed enrichment of *let-7*, *mir-100* and *mir-60* in the vesicle rather than supernatant fraction [9]. On the other hand, *hco-miR-5960-5p*, *hco-miR-45-3p*, *hco-miR-5352-3p*, *hco-miR-87a-3p* and *hco-miR-84a-5p* are all enriched in the L4 EV-depleted libraries.

### Correlating ES miRNA expression with developmental stage and gut localisation

The data in Table 3 highlighted differences in the expression profile of miRNAs between the adult total ES and the adult whole worm libraries with regard to read numbers. However, this table does not show the changes in the miRNA abundance across life stages or their presence in gut tissue. It was therefore of interest to determine if the expression of miRNAs in the ES correlated with their developmental expression in different life-cycle stages and gut localisation.

The data from the small RNA libraries were cross-referenced with the microarray data generated previously. Each library was sorted by maximum reads and the ten most abundant miRNAs were selected and compared to the microarray signal. A number of miRNAs did not have corresponding microarray data as they are not considered as miRNAs and are not in the miRBase database and were excluded from the analysis.

S4 Table shows the ten miRNAs that were most highly expressed in the adult total ES library. *hco-miR-5960-5p* and *3p* were the first and second most abundant, respectively, and both are more highly expressed in the adult male compared to the female. Both versions of *hco-miR-5960* are highly expressed in the *H*. *contortus* gut, dissected from female worms. *hco-miR-5960-3p* also differs slightly from the 5p version by being highly expressed in the L4 stage as well. Of the remaining eight miRNAs, only *hco-miR-228-5p* was highly expressed in the L3 stage, the remaining miRNAs having low expression in the L3 stage. These eight miRNAs also showed higher expression in the adult female compared to the male and were not abundant in the gut tissue. Overall, this suggests that most of the abundant miRNAs present in the adult worm total ES are not released from the gut. A similar pattern was observed for the ten most abundant miRNAs in the adult EV-enriched or adult EV-depleted supernatant (S5 Table and S6 Table, respectively). In contrast, 40–50% of the top ten miRNAs in the L4 EV and EV-depleted libraries were enriched in gut tissue (S7 Table and S8 Table, respectively)

### The related nematode *Teladorsagia circumcincta* also releases miRNAs in EV

To investigate whether EV containing miRNAs were also present in a nematode closely related to *H*. *contortus*, EV were purified from the ES of the L4 stage of *T*. *circumcincta* exactly as described previously [16], and a small RNA library generated, as described for *H*. *contortus*. The absence of any available *T*. *circumcincta* miRNA data necessitated mapping reads from the small RNA library to the entire miRBase database, including *H*. *contortus* sequences (miRBase release 21). BLAST was used to query the miRBase mature sequence database with the reads from the *T*. *circumcincta* small RNA library. This method identified 13,637 miRNAs across 190 different species.

S9 Table shows a comparison of the miRNAs found in *T*. *circumcincta* L4 EV and *H*. *contortus* L4 EV. It is notable that of the top 20 most abundant *T*. *circumcincta* EV miRNAs, 14 were found in the *H*. *contortus* L4 EV library. Interestingly a number of these (asu-*miR-100a-5p*, *miR-60-3p*, *miR-71-5p*, *let-7-5p*, *lin-4*-5p and *miR-5885a*,*b*,*c-3p*) were also present in EV libraries prepared from *H*. *polygyrus* adult stage [9], while *miR-100a-5p* and *let-7-5p* were recently identified in adult EV of the clade I nematode *Trichuris muris* [21]. This suggests there is some conservation of the repertoire of nematode miRNAs packaged into EVs.

## Discussion

In this study we characterised a cluster of four closely linked miRNAs from the pathogenic nematode *H*. *contortus*. The *miR-5352* cluster was shown to be present in an additional ten parasitic nematodes, nine of which were clade V species. None of these sequences had previously been reported as miRNAs and none are included in miRBase, with the exception of the *Ascaris miR-5352* and *miR-43* sequence*s*. The genome of this clade III species contained only *miR-5352* and *miR-43*, based on current sequence data, as did *T*. *canis*, consistent with its close relationship to *Ascaris*. However, the cluster is not present in other clade III nematodes, such as *Brugia spp*. nor is it present in free-living clade V nematodes such as *C*. *elegans*. As the presence of the cluster was not related to nematode clade, we hypothesised that it may be associated with the anatomical site in which the adult worm lives. A common feature of all the nematodes that possess the *miR-5352* cluster is that the adult parasites are not tissue dwelling but reside in the gastro-intestinal tract of the host, with the exception of the bovine lungworm, *D*. *viviparus*. The adult worms of this species parasitize the lungs of the bovine host, although the larvae do traverse the intestine. These results indicate that the presence of the cluster may relate to life in the gut or at a mucosal site. Of the nematodes that possess the *miR-5352* cluster, both *H*. *polygyrus* and *T*. *circumcincta* have multiple versions of the *miR-5352* cluster. The high similarity between these sequences and their proximity in the genome suggest that they may have arisen through gene duplication and divergence, the significance of which is currently unknown. Alternatively, the apparent presence of multiple copies of the cluster may be explained by poor genome assembly, which can be verified from analysis of newer assemblies as these become available.

From the *H*. *contortus* transcript microarray and qRT-PCR data [5], the pattern of expression of each of the miRNAs in the *hco-miR-5352* cluster was very similar: all four were expressed at low levels in the larval stages, rising dramatically in the adult male and female worms. These data imply that the cluster is likely to be co-ordinately expressed and may indicate a stage-specific function for these small RNAs within adult worms or in interacting with their immediate environment. As the presence of the cluster correlates with the anatomical site in the host, it seemed plausible that some of the miRNAs in the *miR-5352*-cluster may be secreted and thus have the potential to interact with host cells. Recent data from studies on other parasitic helminths have shown that miRNAs are frequently present in ES products and can be packaged within EV or exosomes [9,12,16,21–23]. As the gut is thought to be the site from which EV are released in *H*. *polygyrus* worms [9], and is the site of intense metabolic activity in adult *H*. *contortus* [14,24], the gut was dissected from adult female worms and analysed for miRNA expression. While some miRNAs were highly expressed in gut tissue, the four members of the *hco-miR-5352* cluster were not, suggesting that they are unlikely to be gut-derived. However, *miR-5352* could be detected in eggs/hatched L1 of *H*. *contortus* collected from *in vitro* cultures at a similar level to that detected in the whole female worm, suggesting that the embryos or the gonad may be the major source of this miRNA in female worms and possibly also ES.

Initially, a small RNA library was generated from the ES products of mixed sex adult *H*. *contortus* and the small RNA content of the worm ES library compared with that of the whole adult worm, as published previously [5]. A much higher percentage of reads in the ES library mapped to ribosomal RNAs than from the adult worm library, a feature that has been noted in other helminth ES libraries [9]. In addition, in *S*. *mansoni* ES products, Nowacki *et al*., [12] identified a class of tRNA-derived small RNAs, which were hypothesised to be a possible source of additional small non-coding RNAs. These tRNAs fragments were derived from mature tRNAs and could inhibit protein synthesis by interfering with the cap binding complex eIF4F [25].

Direct comparison of the miRNA sequences and their relative abundance in adult worms and their ES products showed that all four members of the *miR-5352* cluster appeared in the top ten miRNAs in adult worm ES indicating that these are secreted *in vitro*. However, there were clear differences in the abundance of miRNAs in adult extract and those released in ES. These data suggest that there may be selectivity in the release of miRNAs by adult worms and that some miRNAs could have the potential to interact directly with host cells rather than functioning within the parasite.

To determine whether miRNAs of the *miR-5352* cluster may be released during infection, tissues from the abomasum epithelium and draining lymph nodes were analysed for the presence of *hco-miR-5352* and *hco-miR-5895*, as well as *hco-miR-5960*, a miRNA that, while not a cluster member, is abundantly secreted *in vitro*. Elevated levels of both cluster miRNAs were detected in *ex vivo* tissue from *H*. *contortus* infected sheep, indicating that these miRNAs are secreted *in vivo* and that they have the potential to interact with and influence the function of cells within both the site of infection and regional lymph nodes. In contrast *hco-miR-5960* could not be specifically detected, suggesting that it may not be secreted *in vivo*, or, alternatively, that the levels of secretion *in vivo* are too low to detect. The apparent enrichment of *hco-miR-5352* in the draining lymph node compared to the abomasal tissue is consistent with the filtering action of the lymph node.

Ultrastructural analysis showed that adult *H*. *contortus* ES contained many small vesicles, consistent in size and appearance with the extracellular vesicles described from other helminth parasite ES products [9,16], although in the present study these were not further characterised. Tzelos *et al*., [16] showed that the EV fraction from L4 *T*. *circumcincta* was recognised by serum from infected animals, implying that EV are secreted *in vivo*. The presence of miRNAs in EV from *H*. *contortus* ES was confirmed by sequencing small RNA libraries prepared from EV-enriched or EV-depleted ES from adult or L4 worms. Hierarchical clustering analysis clearly differentiated groups of miRNAs that were enriched in one population or another. While the EV-enriched library from the L4 stage contained fewer miRNA reads than the EV-depleted fraction, certain miRNAs were more abundant in the L4 EV fraction, perhaps indicating a role in the L4 stage. At day 7 p.i., the L4 are closely apposed to the gastric glands of the abomasum, where the parasite may have direct access to host tissues. Studies on the release of EV from the L3 and adult stages of *B*. *malayi* showed that L3 secrete an abundance of EV *in vitro* compared to adult worms, indicating that EV release may be preferentially associated with larval stages, at least in *Brugia* [11]. However, further comparative studies are required to better understand stage-specific release of both EV and miRNAs in different life cycle stages of *H*. *contortus*.

It is interesting to speculate as to the source of miRNAs, and EV, within the worm. Although none of the four miRNAs of the *miR-5352* cluster were enriched in female gut tissue, it was notable that of the ten most abundant miRNAs present in either L4 EV-enriched or L4 EV-depleted, most were highly expressed in adult worm gut tissue. This observation suggests that these abundantly secreted miRNAs may be released from the worm intestine, as suggested previously for *H*. *polygyrus* EV [9]. L4 stages and particularly adult *H*. *contortus* worms are voracious blood feeders and EV may be released by regurgitation while feeding. We identified a number of miRNAs common to EVs of *H*. *contortus* and *T*. *circumcincta*, and a subset of these was also present in EVs of *H*. *polygyrus* [9], as well as *Brugia* L3 larvae [11]. This observation suggests evolutionary conservation for release of specific miRNAs. Interestingly, some of these (*let-7*, *miR-100*) have identity within the seed region to mammalian miRNAs, implying that they could potentially regulate host genes [21], or alternatively compete in host miRNA-mediated regulation.

In conclusion, the data presented here support the hypothesis that selected miRNAs are released by parasitic nematodes both within EV and free of EV. Further studies are required to elucidate how miRNAs are secreted, particularly those not associated with EV. Are these in complexes with an Argonaute or otherwise stabilised by association with other proteins as has been observed in serum, plasma, and other body fluids [26,27]. For example, miRNAs have been identified in plasma, in fractions of high-density lipoprotein (HDL). Furthermore, HDL can deliver miRNAs to recipient cells and lead to altered gene expression [28]. The detection of two *H*. *contortus* miRNAs in host tissue provides further evidence of a possible role for miRNAs in parasitism, at least for those parasites that inhabit the gut. Further studies with *H*. *contortus* are underway to establish how miRNAs are released by the parasite and whether they have the ability to interact with host cells to influence expression of immune response genes, as described for *H*. *polygyrus*.

## Supporting information

S1 Fig Alignment of the three *T*. *circumcincta miR-5352* cluster sequences obtained from WormBase ParaSite. Highlighted nucleotides are divergent and dashes (-) indicate gaps in the alignment. The purple bars indicate the miRBase precursor sequence and the red bars indicate the miRBase mature sequence.
(TIF)

S2 Fig Multiple alignment of the *Hco-miR-5352* and *Hco-miR-43* precursor and mature sequence and *Ascaris suum miR-5352* and *miR-43* sequences against the *A*. *suum* genome scaffold (scaffold:AscSuum_1.0_submitted:Scaffold187:163878:164556:-1) and A. lumbricoides genome scaffold (A_lumbricoides_Ecuador_v1_5_4, scaffold ALUE_scaffold0000721). Highlighted nucleotides show divergence in the alignment and dashes (-) indicate gaps. The red bars indicate the miRBase mature sequences and the pink bars indicate precursor sequences.
(TIF)

S3 Fig Multiple alignment of the *Dictyocaulus viviparus* contig 497 with the precursor and mature sequences of the *H*. *contortus Hco-miR-5352* cluster. Highlighted nucleotides show divergence between the *D*. *viviparus* sequence and the *H*. *contortus* sequences. Dashes (-) indicate gaps between the alignment.
(TIF)

S4 Fig RNA folding *of miR-5352* homologues in *D*. *viviparus* using Mfold (http://unafold.rna.albany.edu/?q=mfold). (TIF)

S5 Fig Phylogenetic tree of the *Hco-miR-5352* cluster sequences obtained from BLAST analysis. Sequences were only identified in the sub-order Strongylida, but not in the super-family Metastrongyloidea. The tree is drawn to scale, with branch lengths, a measure of the divergence between two nodes in a tree, expressed as substitutions per site of the sequence alignment.
(TIF)

S6 Fig qRT-PCR amplification to detect presence of *oar-miR-26a* in infected and pathogen-free sheep abomasal and draining lymph node tissus. Results show the mean ± SE of three technical replicates with values shown as fluorescence.
(TIFF)

S1 Table Nucleotide sequence of qRT-PCR primers used. *Oar-miR-122a*, *Oar-miR-26a* and *Oar-miR-103* were adapted from their bovine homologues [15], based on the ovine sequence available at NCBI (oar_v3.1). (DOCX)

S2 Table Nematode species and locations of *miR-5352* cluster homologues. (DOCX)

S3 Table MirBase accepted normalised microRNA reads in the EV and EV-depeleted ES libraries. microRNAs that had less than 10 reads in two or more of the libraries were discarded from the final list. miRNAs shown in bold indicate those present in the *Hco-miR-5352* cluster. microRNAs are ordered by miRNA number.
(DOCX)

S4 Table The expression profile across life cycle stages for the ten most abundant miRNAs in the adult total ES. Numbers indicate the normailsed read counts from miRNA microarray analysis of *H*. *contortus* worm stage/tissue. Red indicates high abundance, blue low abundance.
(DOCX)

S5 Table The expression profile across life cycle stages for the ten most abundant miRNAs in the adult EV-enriched supernatant. (DOCX)

S6 Table The expression profile across life cycle stages for the ten most abundant miRNAs in the adult EV-depleted fraction. (DOCX)

S7 Table The expression profile across life cycle stages for the ten most abundant miRNAs in the L4 EV-enriched supernatant. (DOCX)

S8 Table The expression profile across life cycle stages for the ten most abundant miRNAs in the L4 EV-depleted libraries. (DOCX)

S9 Table Comparison between the *T*. *circumcincta* and *H*. *contortus* L4 EV miRNA reads. Numbers indicate the normalised read numbers from the respective libraries. Data shown if the reads in one library exceeded the cut-off of 10.
(DOCX)
