# Supplementary figures and images for: Conservation of a microRNA cluster in parasitic nematodes and profiling of miRNAs in excretory-secretory products and microvesicles of Haemonchus contortus
Source: PLoS Negl Trop Dis. 2017 Nov 16;11(11):e0006056. doi: 10.1371/journal.pntd.0006056 (PMC5709059; doi:10.1371/journal.pntd.0006056)

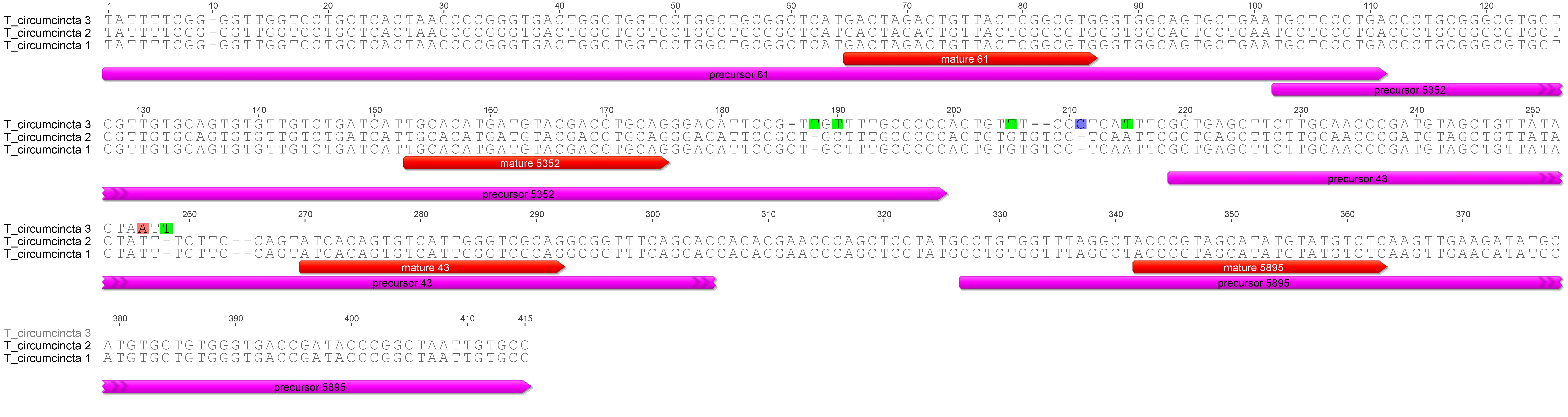

Supplement: S1 Fig — Highlighted nucleotides are divergent and dashes (-) indicate gaps in the alignment. The purple bars indicate the miRBase precursor sequence and the red bars indicate the miRBase mature sequence. (TIF) [file pntd.0006056.s001.tif]

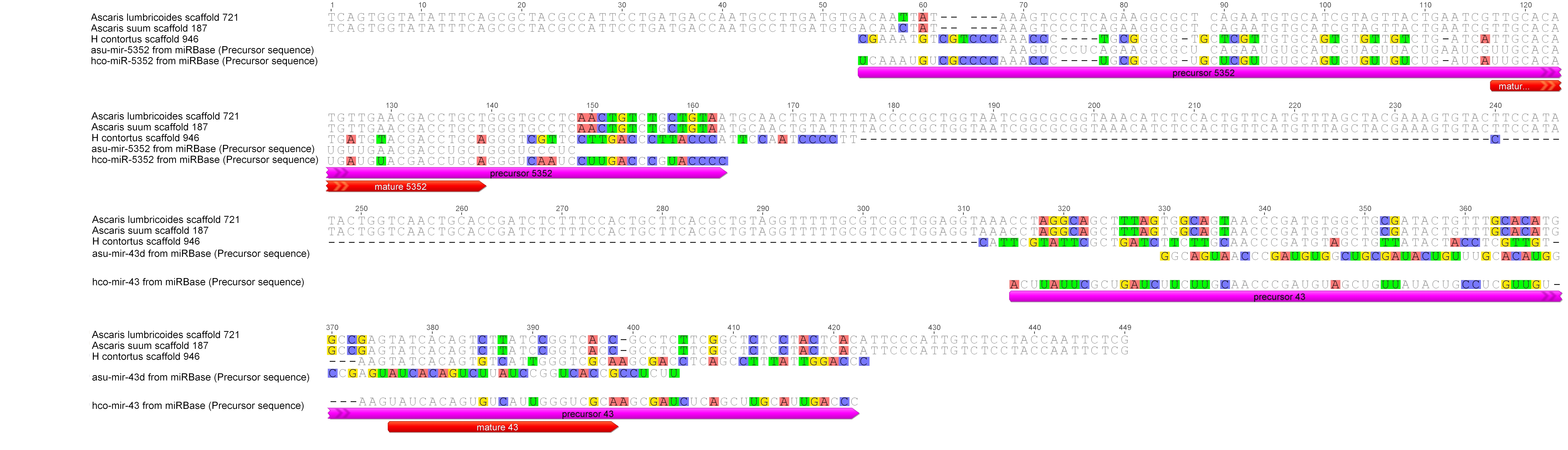

Supplement: S2 Fig — Highlighted nucleotides show divergence in the alignment and dashes (-) indicate gaps. The red bars indicate the miRBase mature sequences and the pink bars indicate precursor sequences. (TIF) [file pntd.0006056.s002.tif]

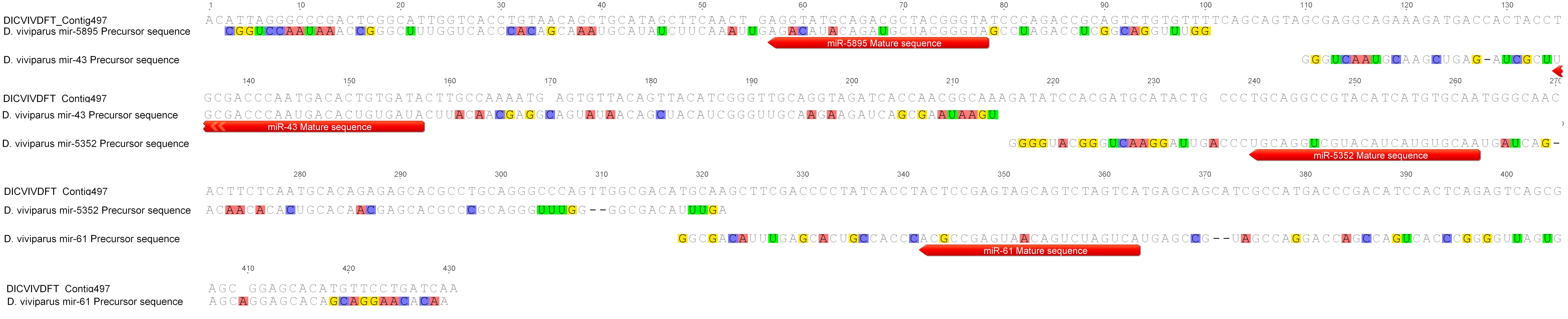

Supplement: S3 Fig — Highlighted nucleotides show divergence between the D. viviparus sequence and the H. contortus sequences. Dashes (-) indicate gaps between the alignment. (TIF) [file pntd.0006056.s003.tif]

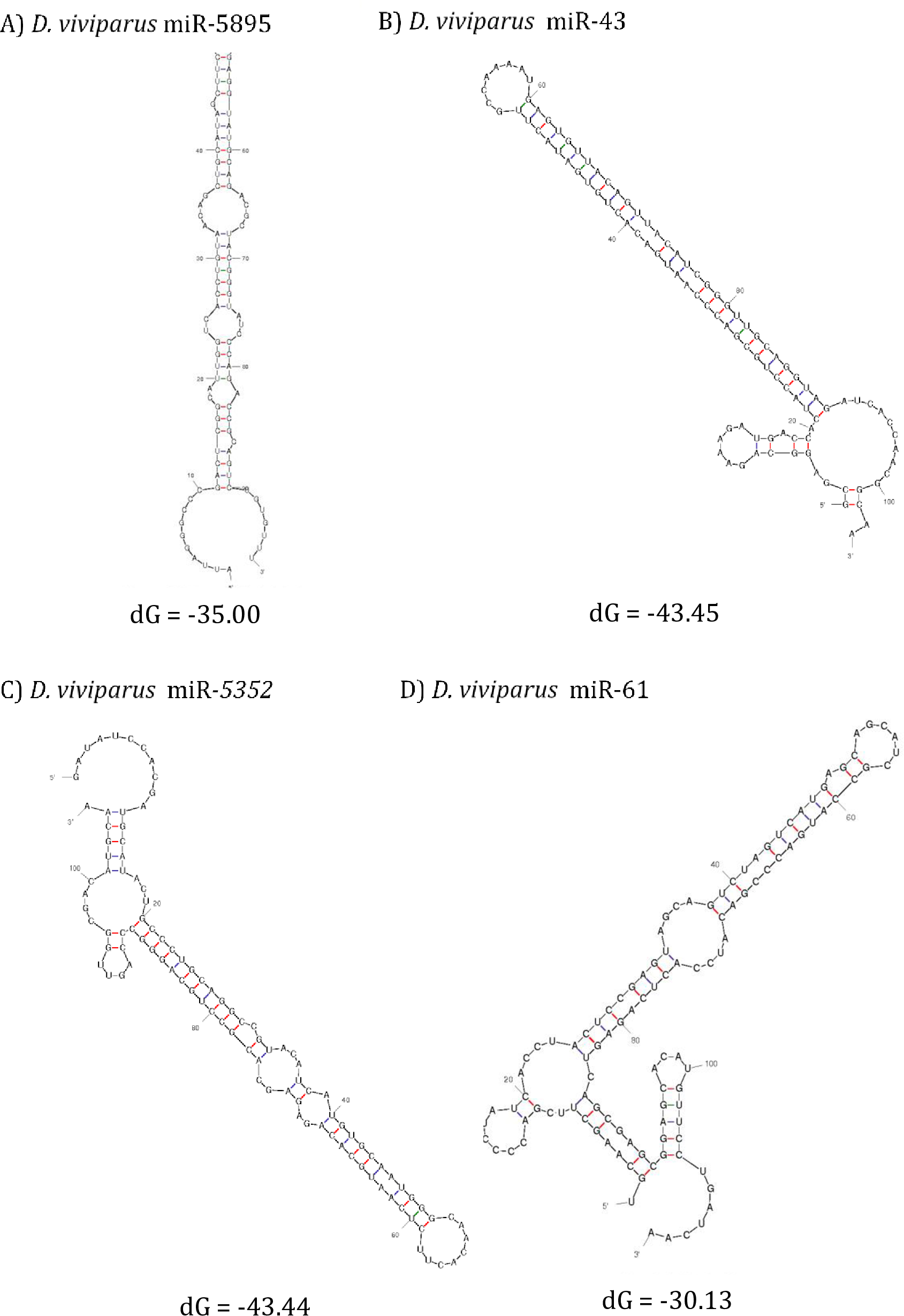

Supplement: S4 Fig — (TIF) [file pntd.0006056.s004.tif]

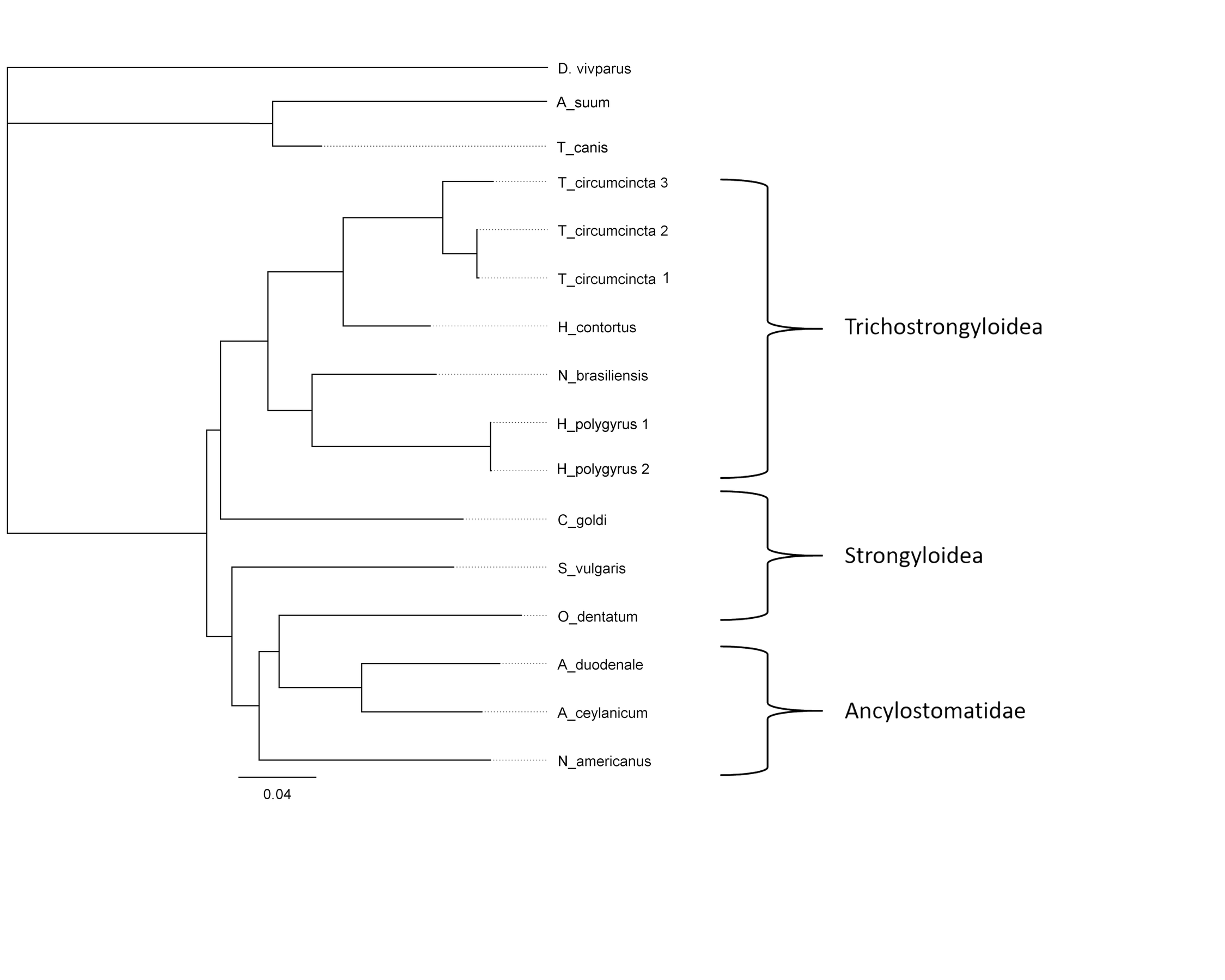

Supplement: S5 Fig — Sequences were only identified in the sub-order Strongylida, but not in the super-family Metastrongyloidea. The tree is drawn to scale, with branch lengths, a measure of the divergence between two nodes in a tree, expressed as substitutions per site of the sequence alignment. (TIF) [file pntd.0006056.s005.tif]

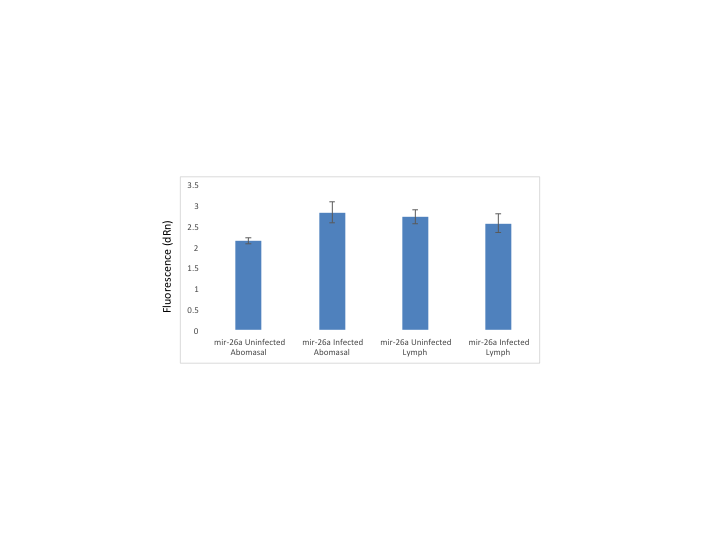

Supplement: S6 Fig — Results show the mean ± SE of three technical replicates with values shown as fluorescence. (TIFF) [file pntd.0006056.s006.tiff]
