# Supplementary material for: Conservation of a microRNA cluster in parasitic nematodes and profiling of miRNAs in excretory-secretory products and microvesicles of Haemonchus contortus
Source: PLoS Negl Trop Dis. 2017 Nov 16;11(11):e0006056. doi: 10.1371/journal.pntd.0006056 (PMC5709059; doi:10.1371/journal.pntd.0006056)
Supplement: S1 Table — (DOCX) [file pntd.0006056.s007.docx]

| miRNA | miRBase ID | Sequence |
| --- | --- | --- |
| *Hco-miR-5895* | MIMAT0023337 | CGT AGC ATC TGT ATG TCT |
| *Hco-miR-5960* | MIMAT0023464 | GGA GTC GGA GGG TTA T |
| *Hco-miR-5352* | MIMAT0023381 | TTG CAC ATG ATG TAC GAC C |
| *Oar-miR-122a* | Adapted from Bta-miR-122a  MIMAT0003849 | TGG AGT GTG ACA ATG GTG |
| *Oar-miR-26a* | Adapted from Bta-miR-26a  MIMAT0003516 | TTC AAG TAA TCC AGG ATA GG |
| *Oar-miR-103* | Adapted from Bta-miR-103  MIMAT0003521 | AGC AGC ATT GTA CAG GG |
