# Supplementary material for: Conservation of a microRNA cluster in parasitic nematodes and profiling of miRNAs in excretory-secretory products and microvesicles of Haemonchus contortus
Source: PLoS Negl Trop Dis. 2017 Nov 16;11(11):e0006056. doi: 10.1371/journal.pntd.0006056 (PMC5709059; doi:10.1371/journal.pntd.0006056)
Supplement: S2 Table — (DOCX) [file pntd.0006056.s008.docx]

| **Nematode** | **Host** | **Site of infection** | **BioProject ID** | **Source** | **Reference if published** |
| --- | --- | --- | --- | --- | --- |
| ***Haemonchus contortus*** | Sheep | Abomasum | PRJEB506 | scaffold_946:77356-77781 | (Laing *et al.,* 2013) |
|  |  |  | PRJNA205202 | scaffold3018:70981-71406 | (Schwarz et al., 2013) |
| ***Ascaris suum*** | Pig | Small intestine | [PRJNA80881](http://www.ebi.ac.uk/ena/data/view/PRJNA80881) | Scaffold187:163878-164318 | (Wang *et al.*, 2012) |
|  |  |  | [PRJNA62057](http://www.ebi.ac.uk/ena/data/view/PRJNA62057) | ALUE_scaffold0000721:20419-20859 | (Jex *et al.,* 2011) |
| ***Oesophagostomum dentatum*** | Pig | Large intestine, caecum | PRJNA72579 | OESDENDFT_Contig26858:966-1532 |  |
| ***Nippostrongylus brasiliensis*** | Rat | Small intestine | PRJEB511 | NBR_scaffold0000464:48929-49370 |  |
| ***Necator americanus*** | Human | Intestine | PRJNA72135 | KI658292:31795-32338 | (Tang *et al.,* 2014) |
| ***Strongylus vulgaris*** | Equine | Large intestine | PRJEB531 | SVUK_scaffold0021520:1240-1820 |  |
| ***Heligmosomoides polygyrus/ (bakeri)*** | Woodmouse and small rodent | Duodenum and small intestine | PRJEB1203 | HPBE_scaffold0001328:47993-48493 |  |
|  |  |  | PRJEB15396 | nHp.2.0.scaf00303:246841-247341 |  |
| ***Cylicostephanus goldi*** | Equine | Large intestine | PRJEB498 | CGOC_contig0000369:2551-3098 |  |
| ***Ancylostoma duodenale*** | Human | Intestine | PRJNA72581 | ANCDUODFT_Contig1102:20935-21505 |  |
| ***Ancylostoma ceylaniucm*** | Human and hamsters | Intestine | PRJNA231479 | Acey_s0007_scaf:1479546-1480112 | (Schwarz *et al.*, 2015) |
|  |  |  | PRJNA72583 | ANCCEYDFT_Contig956:64789-65355 |  |
| ***Dictyocaulus viviparus*** | Bovine | Lung | PRJNA72587 | nDv.1.0.scaf00709:30824-31249 | (McNulty *et al.*, 2016) |
| ***Teladorsagia circumcincta*** | Sheep | Abomasum | PRJNA72569 | TELCIRDFT_Contig190:144482:145265 |  |
|  |  |  |  | TELCIRDFT_Contig190:144694:145491 |  |
