# Supplementary material for: Conservation of a microRNA cluster in parasitic nematodes and profiling of miRNAs in excretory-secretory products and microvesicles of Haemonchus contortus
Source: PLoS Negl Trop Dis. 2017 Nov 16;11(11):e0006056. doi: 10.1371/journal.pntd.0006056 (PMC5709059; doi:10.1371/journal.pntd.0006056)
Supplement: S3 Table — microRNAs that had less than 10 reads in two or more of the libraries were discarded from the final list. miRNAs shown in bold indicate those present in the Hco-miR-5352 cluster. microRNAs are ordered by miRNA number. (DOCX) [file pntd.0006056.s009.docx]

| **MicroRNA** | **Adult EV-depleted** | **Adult EV** | **L4 EV-depleted** | **L4 EV** |
| --- | --- | --- | --- | --- |
| *asu-miR-100a-5p* | 5212 | 3181 | 2463 | 55492 |
| *bma-mir-36b-5p* | 0 | 12 | 12 | 31 |
| *cbn-mir-64f-5p* | 0 | 20 | 13 | 72 |
| *cel-let-7-5p* | 34 | 596 | 1041 | 13532 |
| *cel-miR-50-5p* | 37 | 96 | 135 | 198 |
| *cel-mir-5592-1-3p* | 7 | 26 | 4 | 120 |
| *Hco-lin-4-5p* | 62 | 614 | 4092 | 11107 |
| *Hco-miR-228-5p* | 163 | 48 | 66 | 258 |
| *Hco-miR-236-3p* | 3 | 269 | 31 | 503 |
| *Hco-miR-2-3p* | 30 | 13 | 66 | 123 |
| *Hco-miR-250-3p* | 30 | 14 | 6 | 3 |
| *Hco-miR-259-5p* | 1 | 129 | 53 | 327 |
| *Hco-miR-40b-3p* | 541 | 531 | 3 | 0 |
| ***Hco-miR-43-3p*** | 659 | 157 | 2 | 3 |
| ***Hco-miR-43-5p*** | 795 | 457 | 12 | 50 |
| *Hco-miR-45-3p* | 1160 | 1681 | 1567 | 179 |
| ***Hco-miR-5352-3p*** | 720 | 462 | 2428 | 31 |
| *Hco-miR-5884-5p* | 25 | 122 | 17 | 113 |
| *Hco-miR-5885a-3p* | 16 | 664 | 260 | 4347 |
| *Hco-miR-5885b-3p* | 28 | 672 | 113 | 1058 |
| *Hco-miR-5885c-3p* | 12 | 378 | 114 | 1356 |
| ***Hco-miR-5895-5p*** | 2267 | 2289 | 78 | 82 |
| *Hco-miR-5899-3p* | 1678 | 1991 | 5606 | 4565 |
| *Hco-miR-5908-3p* | 20 | 936 | 111 | 1009 |
| *Hco-miR-5939-3p* | 166 | 36 | 7 | 126 |
| *Hco-miR-5960-3p* | 405 | 115 | 63 | 186 |
| *Hco-miR-5960-5p* | 4070 | 638 | 17207 | 1361 |
| *Hco-miR-5976-5p* | 11 | 144 | 40 | 28 |
| *Hco-miR-60-3p* | 2 | 35 | 16 | 53 |
| ***Hco-miR-61-3p*** | 924 | 422 | 3 | 0 |
| *Hco-miR-63a-3p* | 393 | 187 | 81 | 104 |
| *Hco-miR-63b-3p* | 461 | 307 | 113 | 129 |
| *Hco-miR-71-5p* | 87 | 282 | 355 | 317 |
| *Hco-miR-83-3p* | 19 | 346 | 341 | 2341 |
| *Hco-miR-84a-5p* | 3 | 250 | 319 | 66 |
| *Hco-miR-87a-3p* | 57 | 20 | 160 | 25 |
| *Hco-miR-9551-3p* | 308 | 178 | 18 | 0 |
| *Hco-miR-993-3p* | 6 | 9 | 20 | 25 |
| *prd-miR-7911a-5p* | 0 | 24 | 28 | 187 |
| *prd-miR-7911c-5p* | 17 | 38 | 13 | 195 |
