# Supplementary material for: Conservation of a microRNA cluster in parasitic nematodes and profiling of miRNAs in excretory-secretory products and microvesicles of Haemonchus contortus
Source: PLoS Negl Trop Dis. 2017 Nov 16;11(11):e0006056. doi: 10.1371/journal.pntd.0006056 (PMC5709059; doi:10.1371/journal.pntd.0006056)
Supplement: S4 Table — Numbers indicate the normailsed read counts from miRNA microarray analysis of H. contortus worm stage/tissue. Red indicates high abundance, blue low abundance. (DOCX) [file pntd.0006056.s010.docx]

| **Adult Total ES** | **L3** | **L3(act)** | **L4** | **Male** | **Female** | **Gut** |
| --- | --- | --- | --- | --- | --- | --- |
| *Hco-miR-5960-5p* | 2725 | 2565 | 2924 | 12404 | 6021 | 20908 |
| *Hco-miR-5960-3p* | 9767 | 8033 | 23777 | 25282 | 13540 | 16081 |
| *Hco-miR-5895-5p* | 1350 | 1220 | 339 | 5683 | 13604 | 1111 |
| *Hco-miR-45-3p* | 3961 | 3736 | 5239 | 2024 | 5900 | 1494 |
| *Hco-miR-61-3p* | 279 | 287 | 67 | 10133 | 25106 | 619 |
| *Hco-miR-228-5p* | 3071 | 2705 | 696 | 466 | 253 | 21 |
| *Hco-miR-43-3p* | 1395 | 1319 | 82 | 10688 | 21793 | 1015 |
| *Hco-miR-5352-3p* | 27 | 24 | 37 | 1656 | 6805 | 130 |
| *Hco-miR-40b-3p* | 43 | 40 | 8 | 13 | 223 | 54 |
| *Hco-miR-5884-5p* | 26 | 34 | 151 | 170 | 11413 | 132 |
