# Supplementary material for: Conservation of a microRNA cluster in parasitic nematodes and profiling of miRNAs in excretory-secretory products and microvesicles of Haemonchus contortus
Source: PLoS Negl Trop Dis. 2017 Nov 16;11(11):e0006056. doi: 10.1371/journal.pntd.0006056 (PMC5709059; doi:10.1371/journal.pntd.0006056)
Supplement: S5 Table — (DOCX) [file pntd.0006056.s011.docx]

| **Adult EV-enriched** | **L3** | **L3(act)** | **L4** | **Male** | **Female** | **Gut** |
| --- | --- | --- | --- | --- | --- | --- |
| *Hco-miR-5960-5p* | 2725 | 2565 | 2924 | 12404 | 6021 | 20908 |
| *Hco-miR-5895-5p* | 1350 | 1220 | 339 | 5683 | 13604 | 1111 |
| *Hco-miR-45-3p* | 3961 | 3736 | 5239 | 2024 | 5900 | 1494 |
| *Hco-miR-5352-3p* | 27 | 24 | 37 | 1656 | 6805 | 130 |
| *Hco-miR-40b-3p* | 43 | 40 | 8 | 13 | 223 | 54 |
| *Hco-miR-5885a-3p* | 341 | 305 | 37413 | 7610 | 14857 | 35869 |
| *Hco-miR-5885b-3p* | 1622 | 1910 | 53743 | 24053 | 29032 | 37793 |
| *Hco-miR-5899-3p* | 11697 | 14170 | 14231 | 13024 | 11559 | 11007 |
| *Hco-miR-5908-3p* | 62 | 55 | 4226 | 13619 | 19883 | 40502 |
| *Hco-lin-4-5p* | 7043 | 8657 | 9419 | 5249 | 6298 | 7551 |
