# Supplementary material for: Conservation of a microRNA cluster in parasitic nematodes and profiling of miRNAs in excretory-secretory products and microvesicles of Haemonchus contortus
Source: PLoS Negl Trop Dis. 2017 Nov 16;11(11):e0006056. doi: 10.1371/journal.pntd.0006056 (PMC5709059; doi:10.1371/journal.pntd.0006056)
Supplement: S6 Table — (DOCX) [file pntd.0006056.s012.docx]

| **A**d**ult EV-depleted** | **L3** | **L3(act)** | **L4** | **Male** | **Female** | **Gut** |
| --- | --- | --- | --- | --- | --- | --- |
| *Hco-miR-5960-5p* | 2725 | 2565 | 2924 | 12404 | 6021 | 20908 |
| *Hco-miR-5960-3p* | 9767 | 8033 | 23777 | 25282 | 13540 | 16081 |
| *Hco-miR-5895-5p* | 1350 | 1220 | 339 | 5683 | 13604 | 1111 |
| *Hco-miR-45-3p* | 3961 | 3736 | 5239 | 2024 | 5900 | 1494 |
| *Hco-miR-61-3p* | 279 | 287 | 67 | 10133 | 25106 | 619 |
| *Hco-miR-43-3p* | 1395 | 1319 | 82 | 10688 | 21793 | 1015 |
| *Hco-miR-5352-3p* | 27 | 24 | 37 | 1656 | 6805 | 130 |
| *Hco-miR-40b-3p* | 43 | 40 | 8 | 13 | 223 | 54 |
| *Hco-miR-5899-3p* | 11697 | 14170 | 14231 | 13024 | 11559 | 11007 |
| *Hco-miR-63b-3p* | 485 | 693 | 452 | 291 | 2775 | 2606 |
