# Supplementary material for: Conservation of a microRNA cluster in parasitic nematodes and profiling of miRNAs in excretory-secretory products and microvesicles of Haemonchus contortus
Source: PLoS Negl Trop Dis. 2017 Nov 16;11(11):e0006056. doi: 10.1371/journal.pntd.0006056 (PMC5709059; doi:10.1371/journal.pntd.0006056)
Supplement: S7 Table — (DOCX) [file pntd.0006056.s013.docx]

| **L4 EV-enriched** | **L3** | **L3(act)** | **L4** | **Male** | **Female** | **Gut** |
| --- | --- | --- | --- | --- | --- | --- |
| *Hco-miR-5960-5p* | 2725 | 2565 | 2924 | 12404 | 6021 | 20908 |
| *Hco-miR-5885a-3p* | 341 | 305 | 37413 | 7610 | 14857 | 35869 |
| *Hco-miR-5885b-3p* | 1622 | 1910 | 53743 | 24053 | 29032 | 37793 |
| *Hco-miR-5885c-5p* | 15 | 13 | 59 | 911 | 8750 | 563 |
| *Hco-miR-5899-3p* | 11697 | 14170 | 14231 | 13024 | 11559 | 11007 |
| *Hco-miR-5908-3p* | 62 | 55 | 4226 | 13619 | 19883 | 40502 |
| *Hco-miR-236-3p* | 41 | 30 | 576 | 55 | 178 | 5114 |
| *Hco-lin-4-5p* | 7043 | 8657 | 9419 | 5249 | 6298 | 7551 |
| *Hco-miR-83-3p* | 1364 | 1231 | 10639 | 3146 | 3240 | 10799 |
| *Hco-miR-1-3p* | 3225 | 3271 | 2411 | 1077 | 541 | 9 |
