# Supplementary material for: Conservation of a microRNA cluster in parasitic nematodes and profiling of miRNAs in excretory-secretory products and microvesicles of Haemonchus contortus
Source: PLoS Negl Trop Dis. 2017 Nov 16;11(11):e0006056. doi: 10.1371/journal.pntd.0006056 (PMC5709059; doi:10.1371/journal.pntd.0006056)
Supplement: S8 Table — (DOCX) [file pntd.0006056.s014.docx]

| **L4 EV-depleted** | **L3** | **L3(act)** | **L4** | **Male** | **Female** | **Gut** |
| --- | --- | --- | --- | --- | --- | --- |
| *Hco-miR-5960-5p* | 2725 | 2565 | 2924 | 12404 | 6021 | 20908 |
| *Hco-miR-45-3p* | 3961 | 3736 | 5239 | 2024 | 5900 | 1494 |
| *Hco-miR-5352-3p* | 27 | 24 | 37 | 1656 | 6805 | 130 |
| *Hco-miR-5885a-3p* | 341 | 305 | 37413 | 7610 | 14857 | 35869 |
| *Hco-miR-71-5p* | 13003 | 18613 | 15138 | 27606 | 25500 | 24902 |
| *Hco-miR-5899-3p* | 11697 | 14170 | 14231 | 13024 | 11559 | 11007 |
| *Hco-lin-4-5p* | 7043 | 8657 | 9419 | 5249 | 6298 | 7551 |
| *Hco-miR-235-3p* | 2762 | 2347 | 80 | 1787 | 1113 | 60 |
| *Hco-miR-83-3p* | 1364 | 1231 | 10639 | 3146 | 3240 | 10799 |
| *Hco-miR-84a-5p* | 6 | 16 | 22187 | 9785 | 10352 | 15138 |
