# Supplementary material for: Conservation of a microRNA cluster in parasitic nematodes and profiling of miRNAs in excretory-secretory products and microvesicles of Haemonchus contortus
Source: PLoS Negl Trop Dis. 2017 Nov 16;11(11):e0006056. doi: 10.1371/journal.pntd.0006056 (PMC5709059; doi:10.1371/journal.pntd.0006056)
Supplement: S9 Table — Numbers indicate the normalised read numbers from the respective libraries. Data shown if the reads in one library exceeded the cut-off of 10. (DOCX) [file pntd.0006056.s015.docx]

|  | *T. circumcincta* L4 EV | *H. contortus* L4 EV |
| --- | --- | --- |
| Hco-miR-235-3p | 3812 | Not Found |
| Hco-miR-45-3p | 2072 | 179 |
| Hco-miR-236-3p | 1331 | 503 |
| Hco-miR-228-5p | 1309 | 258 |
| Hco-miR-72-5p | 1156 | Not Found |
| Hco-miR-5885c-3p | 531 | 1356 |
| Hco-miR-5885a-3p | 528 | 4347 |
| Hco-miR-59-3p | 521 | Not Found |
| asu-miR-100a-5p | 452 | 55492 |
| Hco-miR-5885b-3p | 377 | 1058 |
| Hco-miR-60-3p | 374 | 53 |
| Hco-miR-71-5p | 342 | 317 |
| cel-let-7-5p | 305 | 13532 |
| Hco-lin-4-5p | 298 | 11107 |
| Hco-miR-259-5p | 220 | 327 |
| Hco-miR-5960-5p | 220 | 1336 |
| Hco-miR-993-3p | 201 | 25 |
| Hco-miR-5978-5p | 192 | Not Found |
| Hco-miR-5922-5p | 185 | Not Found |
| Hco-miR-790-5p | 182 | Not Found |
| Hco-miR-5899-3p | 166 | 4565 |
| Hco-miR-50-5p | 157 | Not Found |
| Hco-miR-5991-3p | 144 | Not Found |
| Hco-miR-87a-3p | 104 | 25 |
| Hco-miR-87b-3p | 100 | Not Found |
| Hco-miR-9-5p | 100 | Not Found |
| Hco-miR-84a-5p | 97 | 66 |
| Hco-miR-87c-3p | 91 | Not Found |
| Hco-miR-252-5p | 88 | Not Found |
| Hco-miR-307-3p | 88 | Not Found |
| Hco-miR-63a-3p | 88 | 104 |
| Hco-miR-5908-3p-3p | 82 | Not Found |
| Hco-miR-86-5p | 79 | Not Found |
| Hco-miR-43-3p | 66 | 3 |
| Hco-miR-5948-3p | 66 | Not Found |
| cel-miR-50-5p | 60 | 198 |
| Hco-miR-83-3p | 57 | 2341 |
| Hco-miR-5884-5p | 41 | 113 |
| Hco-miR-79-3p | 41 | Not Found |
| Hco-miR-40b-3p | 31 | Not Found |
| Hco-miR-46-3p | 31 | Not Found |
| Hco-miR-5900-3p | 28 | Not Found |
| Hco-miR-5921-5p-5p | 28 | Not Found |
| bma-mir-36b-p5 | 22 | 31 |
| cbn-mir-64f-p5 | 6 | 72 |
| cel-mir-5592-1-p3 | 3 | 120 |
| prd-miR-7911a-5p | 3 | 187 |
| Hco-miR-2-3p | Not Found | 123 |
| Hco-miR-43-p5 | Not Found | 50 |
| Hco-miR-5352-3p | Not Found | 31 |
| Hco-miR-5895-5p | Not Found | 82 |
| Hco-miR-5908-3p | Not Found | 1009 |
| Hco-miR-5939-3p | Not Found | 126 |
| Hco-miR-5960-p3 | Not Found | 186 |
| Hco-miR-5960-p5 | Not Found | 25 |
| Hco-miR-5976-5p | Not Found | 28 |
| Hco-miR-63b-3p | Not Found | 129 |
| prd-miR-7911c-5p | Not Found | 195 |
